# Optimization and validation of diffusion MRI-based fiber tracking with neural tracer data as a reference

**DOI:** 10.1038/s41598-020-78284-4

**Published:** 2020-12-18

**Authors:** Carlos Enrique Gutierrez, Henrik Skibbe, Ken Nakae, Hiromichi Tsukada, Jean Lienard, Akiya Watakabe, Junichi Hata, Marco Reisert, Alexander Woodward, Yoko Yamaguchi, Tetsuo Yamamori, Hideyuki Okano, Shin Ishii, Kenji Doya

**Affiliations:** 1 Neural Computation Unit, Okinawa Institute of Science and Technology Graduate University, Okinawa, Japan; 2 Brain Image Analysis Unit, RIKEN Center for Brain Science, Wako, Japan; 3 Integrated Systems Biology Laboratory, Department of Systems Science, Graduate School of Informatics, Kyoto University, Kyoto, Japan; 4 Laboratory for Molecular Analysis of Higher Brain Function, RIKEN Center for Brain Science, Wako, Japan; 5 Laboratory for Marmoset Neural Architecture, RIKEN Center for Brain Science, Wako, Japan; 6 Department of Medical Physics and Stereotaxy, Medical Center, Faculty of Medicine, University Freiburg, Freiburg, Germany; 7 Connectome Analysis Unit, RIKEN Center for Brain Science, Wako, Japan; 8 Applied Electronics Laboratory, Kanazawa Institute of Technology, Nonoichi, Japan; 9 Division of Regenerative Medicine, The Jikei University School of Medicine, Tokyo, Japan; 10 Department of Physiology, Keio University School of Medicine, Tokyo, Japan; 11 Graduate School of Information Science and Technology, The University of Tokyo, Tokyo, Japan; 12 Laboratory for Cognitive Brain Mapping, RIKEN Center for Brain Science, Wako, Japan

**Keywords:** Computational neuroscience, Brain

## Abstract

Diffusion-weighted magnetic resonance imaging (dMRI) allows non-invasive investigation of whole-brain connectivity, which can reveal the brain’s global network architecture and also abnormalities involved in neurological and mental disorders. However, the reliability of connection inferences from dMRI-based fiber tracking is still debated, due to low sensitivity, dominance of false positives, and inaccurate and incomplete reconstruction of long-range connections. Furthermore, parameters of tracking algorithms are typically tuned in a heuristic way, which leaves room for manipulation of an intended result. Here we propose a general data-driven framework to optimize and validate parameters of dMRI-based fiber tracking algorithms using neural tracer data as a reference. Japan’s Brain/MINDS Project provides invaluable datasets containing both dMRI and neural tracer data from the same primates. A fundamental difference when comparing dMRI-based tractography and neural tracer data is that the former cannot specify the direction of connectivity; therefore, evaluating the fitting of dMRI-based tractography becomes challenging. The framework implements multi-objective optimization based on the non-dominated sorting genetic algorithm II. Its performance is examined in two experiments using data from ten subjects for optimization and six for testing generalization. The first uses a seed-based tracking algorithm, iFOD2, and objectives for sensitivity and specificity of region-level connectivity. The second uses a global tracking algorithm and a more refined set of objectives: distance-weighted coverage, true/false positive ratio, projection coincidence, and commissural passage. In both experiments, with optimized parameters compared to default parameters, fiber tracking performance was significantly improved in coverage and fiber length. Improvements were more prominent using global tracking with refined objectives, achieving an average fiber length from 10 to 17 mm, voxel-wise coverage of axonal tracts from 0.9 to 15%, and the correlation of target areas from 40 to 68%, while minimizing false positives and impossible cross-hemisphere connections. Optimized parameters showed good generalization capability for test brain samples in both experiments, demonstrating the flexible applicability of our framework to different tracking algorithms and objectives. These results indicate the importance of data-driven adjustment of fiber tracking algorithms and support the validity of dMRI-based tractography, if appropriate adjustments are employed.

## Introduction

Diffusion-weighted magnetic resonance imaging (dMRI) generates images based on anisotropic diffusion of water molecules. Diffusion in the brain is constrained in a direction-dependent manner by obstacles such as nerve fibers and membranes. This leads to anisotropic diffusion patterns in dMRI images that can be used to estimate structural brain connectivity in a non-invasive way^1–5^. dMRI-based tractography can trace whole-brain connectivity to more fully reveal network organization^6–8^, its relationship with functions^9–11^, mental and neurological disorders^12–15^, and computational modeling^16^.

However, there are fundamental limitations, namely, the lack of directionality of connections and the difficulty of estimating crossing fiber orientations in voxels of low spatial resolution^17,18^. These and other practical issues cause failures in tracking fibers (low sensitivity or low true positive rate)^19–21^, especially in tracking long-distance connections^22–24^, and tracking wrong fibers (low specificity or high false positive rate)^20,25,26^. Unfortunately, all of these potentially contribute to erroneous reconstruction of connectomes.

Various efforts have been made to improve the accuracy of reconstructions. Global tractography^27–29^ provides whole-brain connectivity that consistently explains dMRI data by optimizing a global objective function. Compared to conventional seed-based fiber tracking, it achieved better qualitative results on phantom data^27^. However, both seed-based and global fiber tracking algorithms have a number of parameters that are difficult to determine because of unknown biophysical variables.

Japan’s Brain/MINDS project (Brain Mapping by Integrated Neurotechnologies for Disease Studies)^30^ intends to build a multi-scale marmoset brain map and mental disease models. The project has assembled a high-resolution marmoset brain atlas^31^, and is conducting systematic anterograde tracer injections to analyse brain connectivity, while obtaining functional, structural, and diffusion MRI for most individuals. All data are mapped to a common brain space. This gives us a unique opportunity to verify the accuracy of dMRI-based fiber tracking using neuronal tracer data, reconstructed with the marmonet pipeline^32^ as a reference.

Here we propose a general framework for optimization and validation of dMRI-based fiber tracking algorithms in reference to neuronal tracer data from multiple injection sites. Because fiber tracking should satisfy multiple performance criteria, we use multi-objective optimization (MOO) in the first stage and then use multiple criteria decision analysis (MCDA) to select a set of standard parameters. We test the effectiveness of our framework in two experiments. In the first experiment, we use a probabilistic streamline-based algorithm iFOD2^33^ and consider the region-level true positive rate (TPR) and false positive rate (FPR) as criteria. In the second experiment, we take a global tracking algorithm^27^ and incorporate more elaborate criteria: (1) distance-weighted coverage, (2) the true/false positive ratio, (3) projection coincidence, and (4) commissural passage.

We optimize the parameters using 10 brain samples and then test their capacity for generalization using 6 brain samples that were not used for optimization. Our implementation code for processing multiple brain samples in parallel is compatible with HPC (high-performance computing) clusters as well as desktop PCs, and publicly available.

## Results

### Brain/MINDS marmoset connectome data

We use neural tracer data from 20 marmosets collected in the Brain/MINDS project for this study (see Fluorescent neural tracer data at “Methods” section). An anterograde tracer was injected in the left prefrontal cortex, at different points for each animal, and neuron projection pathways as well as their target regions were quantified based on tracer voxel density in fine 500 or coarse 104 parcellation in the Brain/MINDS atlas^31^. We consider an injection region connected to a target region when at least one injection tracer image has signal in both regions. This is the first version of a neural tracer-based connectome computed by the marmonet pipeline^32^ in the project.

For optimization and validation, we took data from 16 animals that had both tracer and dMRI data. Experiments evaluate dMRI-based fiber tracking against multiple objectives, by comparisons with tracer at different levels of resolution: brain region-level and voxel-level. Objectives can be unrelated to tracer. An example of an anatomical constraint is defined as objective in the 2nd experiment.

### Seed-based tracking with region-level criteria

In the first experiment, we take the probabilistic streamline-based algorithm iFOD2^33^ (second-order integration over Fiber Orientation Distributions), which is the default tractography algorithm of MRtrix3^34^. Three important parameters are optimized: (a) *angle*: the maximum angle between successive steps of the algorithm; (b) *cutoff*: the FOD amplitude for terminating fibers; (c) *minlength*: the minimum length, in mm, of any fiber.

The number of seeds (1000 $\times$ number of output fibers) and all other parameters are kept at their default values. Streamline seeds are placed randomly all over the dMRI. The number of output fibers is fixed at 300,000.

#### Criteria for evaluation

An important issue in comparing dMRI-based fiber tracking and anterograde neural tracer data is that the former does not reflect the projection direction. Comparisons assume that regions are connected independently of tracer directionality. dMRI-based fibers connected to a tracer injection site can include both incoming and outgoing axons to the site. Thus, if we take anterograde tracing as a reference, it is natural to have additional “false positive” fibers.

**Figure 1:**
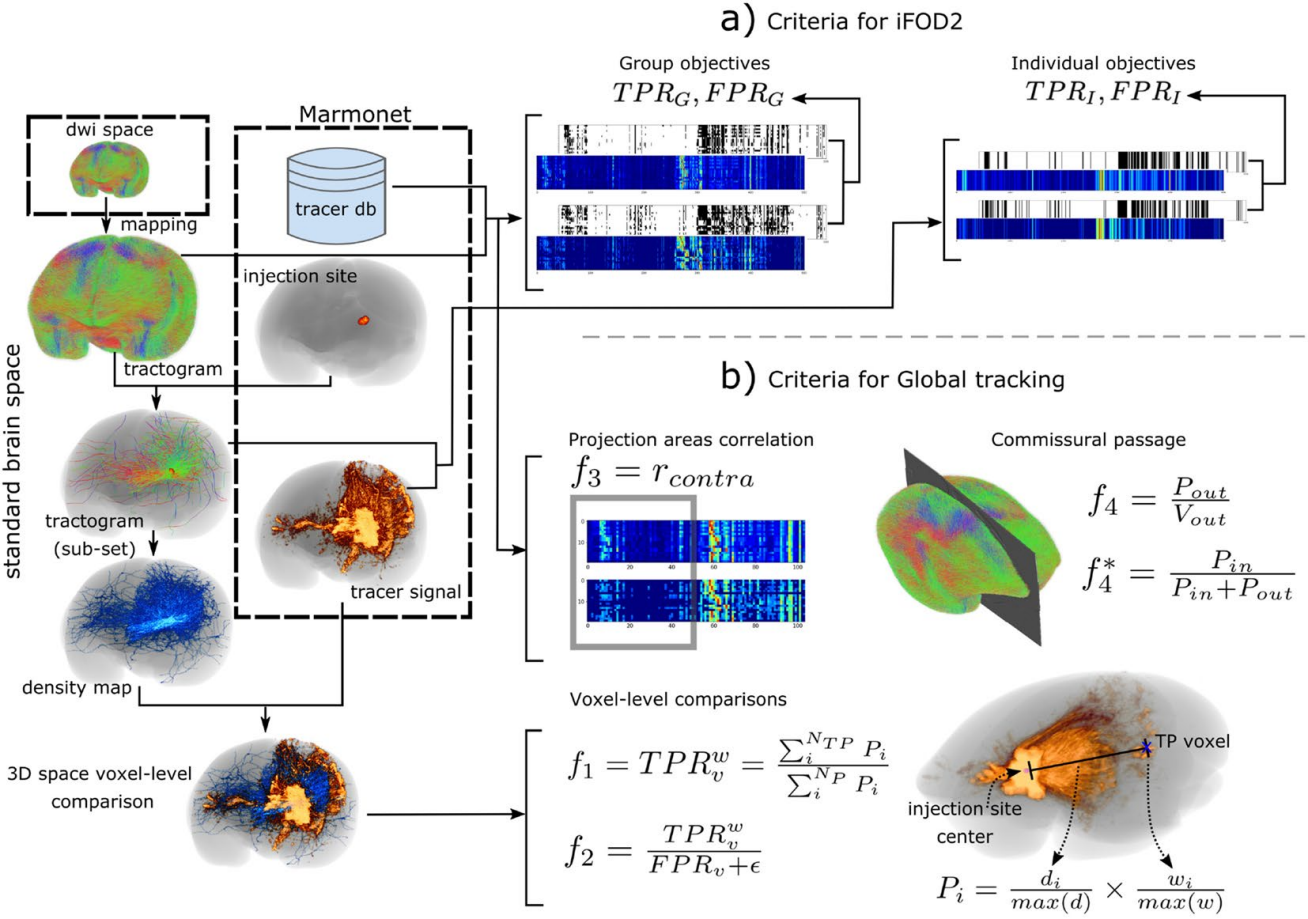
Criteria for evaluation. (**a, b**) show evaluation criteria for the 1st (iFOD2) and 2nd (global tracking) experiments. dMRI-based fiber tracking results are mapped to the standard brain space and intersected spatially with the injection site, allowing extraction of a subset of fibers. The full tractogram is used to compute group $TPR_G$ and $FPR_G$ (iFOD2), projection coincidence with the target hemisphere $f_3$ and the commissural passage $f_4$ (global tracking). The subset of fibers is used for individual $TPR_I$ and $FPR_I$ (iFOD2), the distance-weighted coverage $f_1$ and true/false positive ratio $f_2$ objectives (global tracking). Global tracking includes more elaborated criteria, with positive voxels weighted by two factors extracted from neural tracer data, the distance to the injection site center $d_i$ and the voxel intensity $w_i$. Figure created using The MRtrix viewer 3.0.1 (https://www.mrtrix.org/) and Inkscape 1.0beta2 (https://inkscape.org/). Image datasets are part of the Brain/MINDS project (see Data availability section).

Four objective functions measuring brain-region connectome similarities consider fitting to both individual tracer data and group tracer data in terms of *TP* and *FP* (Fig. 1a). dMRI-based matrices are built for each fiber tracking result in a standard brain space, by assigning each streamline to all regions it intersects. Before comparison, dMRI- and tracer-based matrices are log-transformed and normalized. Matrix binarization, preserving connections from 10 to 100%, is included as a preceding step to *TPR* and *FPR* calculation.

- **Individual objectives** (i) $TPR_I$ and (ii) $FPR_I$. Obtained by comparing individual injection site-region pairs connected by streamlines for each brain. Thus, fibers intersecting the injection region and the tracer of the same animal were arranged as matrices of 1 injection site $\times$ 500 targets parcellation for matching.
- **Group objectives** (iii) $TPR_G$ and (iv) $FPR_G$. Obtained by mapping fiber tracking output to the group of 20 injection sites $\times$ 500 targets parcellation for each brain, and comparing against the Brain/MINDS marmoset connectome data.

#### Multi-objective optimization

In order to account for trade-offs between multiple objectives, instead of optimizing a scalar criterion using the weighted sum of objectives, we took the multi-objective optimization (MOO) approach to find the Pareto-optimal set, or Pareto front, where no objective values can be improved without degrading some other objective values. For our experiment, the non-dominated sorting genetic algorithm II (NSGA-II)^35^ was arranged for parallel optimization of 10 brains (training set). An optimization process runs per brain while, cooperatively, it sends winner parameters to other processes in each generation (see Optimization and Code implementation at “Methods” section).

**Figure 2:**
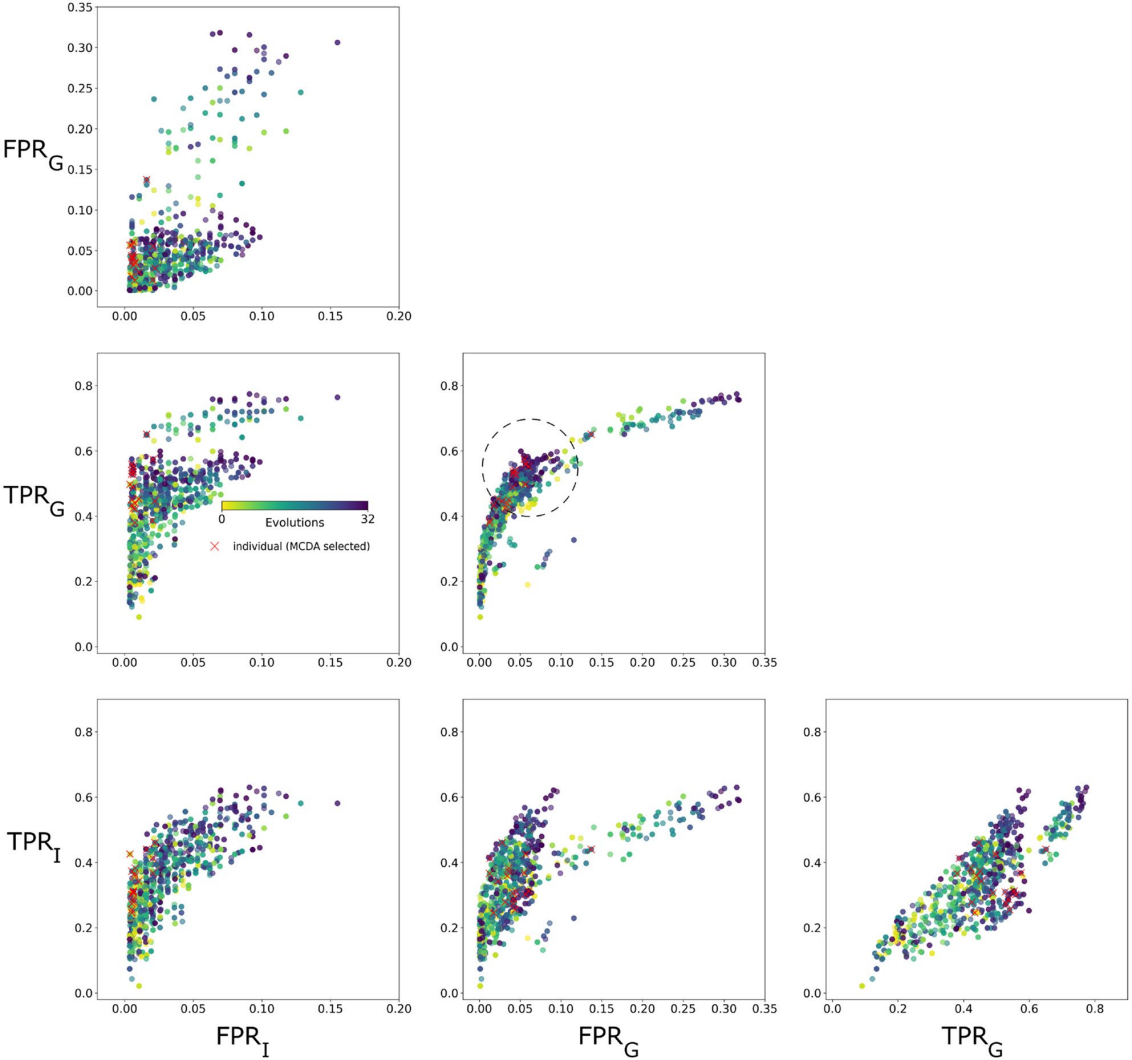
Objective function optimization for iFOD2. Pair-wise visualization of the optimization of four objective functions: $TPR_G$ and $FPR_G$ from the comparison between connectomes of $20 \times 500$, and $TPR_I$ and $FPR_I$ from the comparison between individual connectomes of $1 \times 500$. Our framework drives objectives toward the Pareto-front in the upper-left direction for the competing *TP* versus *FP* objectives. $FPR_G$ versus $TPR_G$ exposes a peak of optimal solutions (dotted circle). $FPR_I$ versus $FPR_G$ evinces the capability of our framework for controlling *FP* growth, maintaining values close to 0, at the bottom-left region. Best solutions, detected by MCDA, are shown as red x markers.

**Figure 3:**
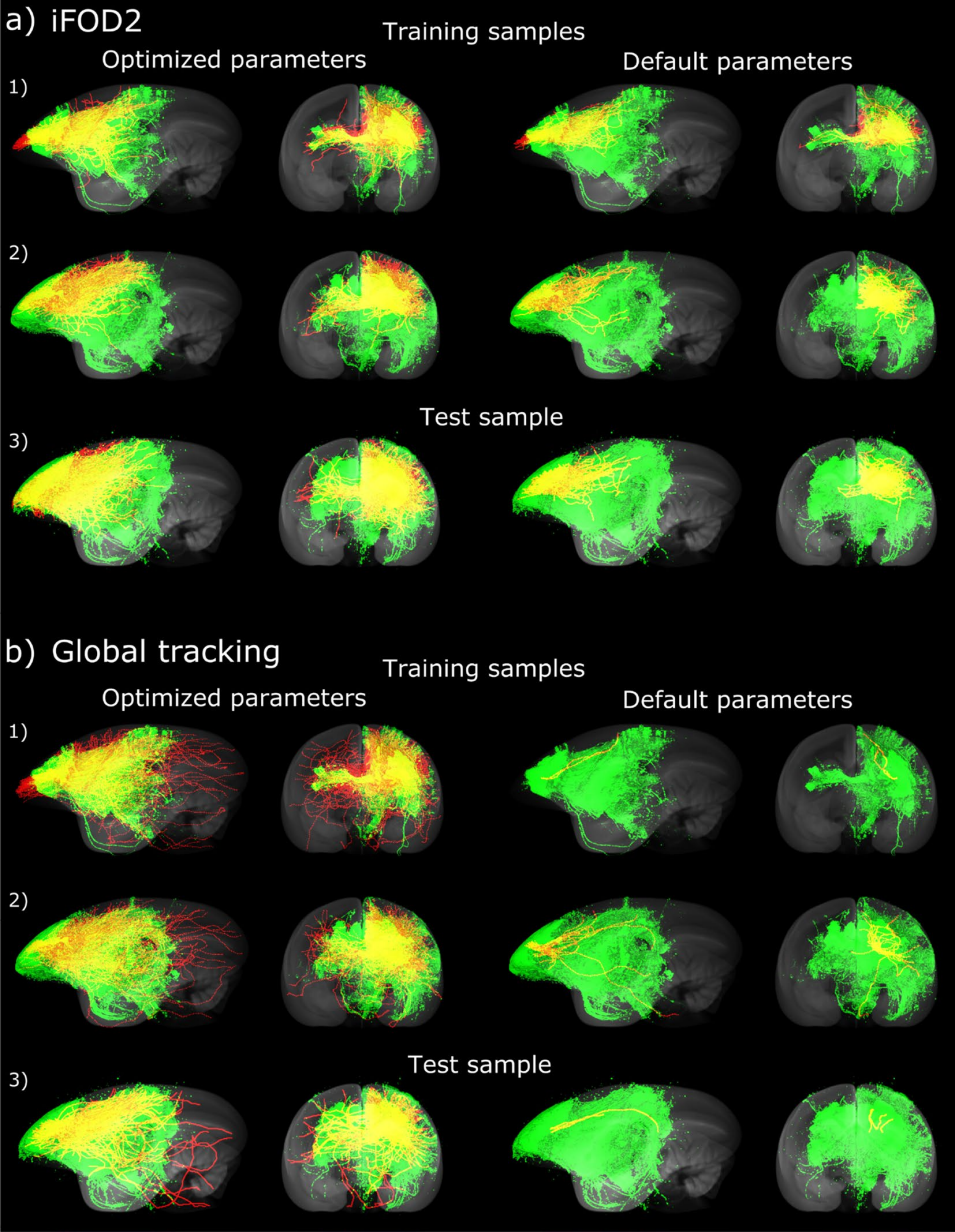
Examples of tracked fibers by optimized and default parameters. Unoccluded visualization of spatial relationships between fluorescent tracer signals (green) and tractography (red) for 3 injection sites: (1, 2) from the training set; (3) from unseen marmoset subjects. Their overlap (yellow) shows common voxels, while red fibers correspond to “false positives”. Improved results for both, (**a**) iFOD2 and (**b**) global tracking algorithms, show enlarged overlap and longer fibers connecting sub-cortical and projection areas. Figure created using FluoRender 2.24 (https://www.sci.utah.edu/software/fluorender.html) and Inkscape 1.0beta2 (https://inkscape.org/). Image datasets are part of the Brain/MINDS project (see Data availability section).

Optimization identified multi-dimensional Pareto fronts, one per brain, which evolved similarly and converged to a common region. They are visualized in Fig. 2 as pairwise comparisons of objectives. The competition of $TPR_G$ versus $FPR_G$ and $TPR_I$ versus $FPR_I$ pushed results toward the upper-left region (ideal region), clearly seen in $TPR_G$ versus $FPR_G$, where the latest evolutionary results peek out from the early made ROC curve (dotted circle). $TPR_G$ versus $FPR_G$ performance suggests that individual brain variability is weakened by connectome-based group objectives. Spatial coverage improved, as seen in Fig. 3a and Supplementary Fig. S1a, where fiber tracking by iFOD2 (in red) covers larger areas of the neural traces (in green) by the optimized parameters. Fiber length increased as well, from a default value of 8.13 mm to an optimized value of around 12.2 mm, on average.

#### Multiple criteria decision analysis for standard parameters

To assess trade-offs between objectives and to determine which combination performs best for each brain (Fig. 2, red x markers) and for the training set, we used Multiple Criteria Decision Analysis (MCDA).

Objectives, denoted as *f*’s, are considered the multiple criteria. Given an optimized brain, each *f* interval [*min*(*f*), *max*(*f*)] is divided into 10 equal sub-intervals and corresponding parameter settings are rated from 1 (worst) to 10 (best). Ratings are averaged across *f*’s with equal weighting for each *f* and brain, and the parameter set with the maximum score is selected as the individual winner(s) for the brain.

An evaluation-averaged result from 5 fiber tracking runs using default parameters for the training set, and compared against the average of individual winners: $TPR_G$ improved from $0.3\pm 0.11$ to $0.5\pm 0.07$ and $TPR_I$ from $0.2\pm 0.09$ to $0.34\pm 0.07$. In the case of *FP* objectives, the optimization kept values down, with no substantial changes: $FPR_G$ from $0.023\pm 0.037$ to $0.04\pm 0.03$ and $FPR_I$ from $0.005\pm 0.006$ to $0.01\pm 0.006$. The restrictive effect of *FP* related objectives is seen in $FPR_I$ versus $FPR_G$ (Fig. 2), where the best solutions are located in the desired bottom-left area.

Standard parameters were calculated as the mean and standard deviation of the best solution parameters for the 10 brains: *angle*: $32.2\pm 6.3$, *cutoff*: $0.05\pm 0.012$, and *minlength*: $4.8\pm 2.5$.

#### Validation by test set data

**Figure 4:**
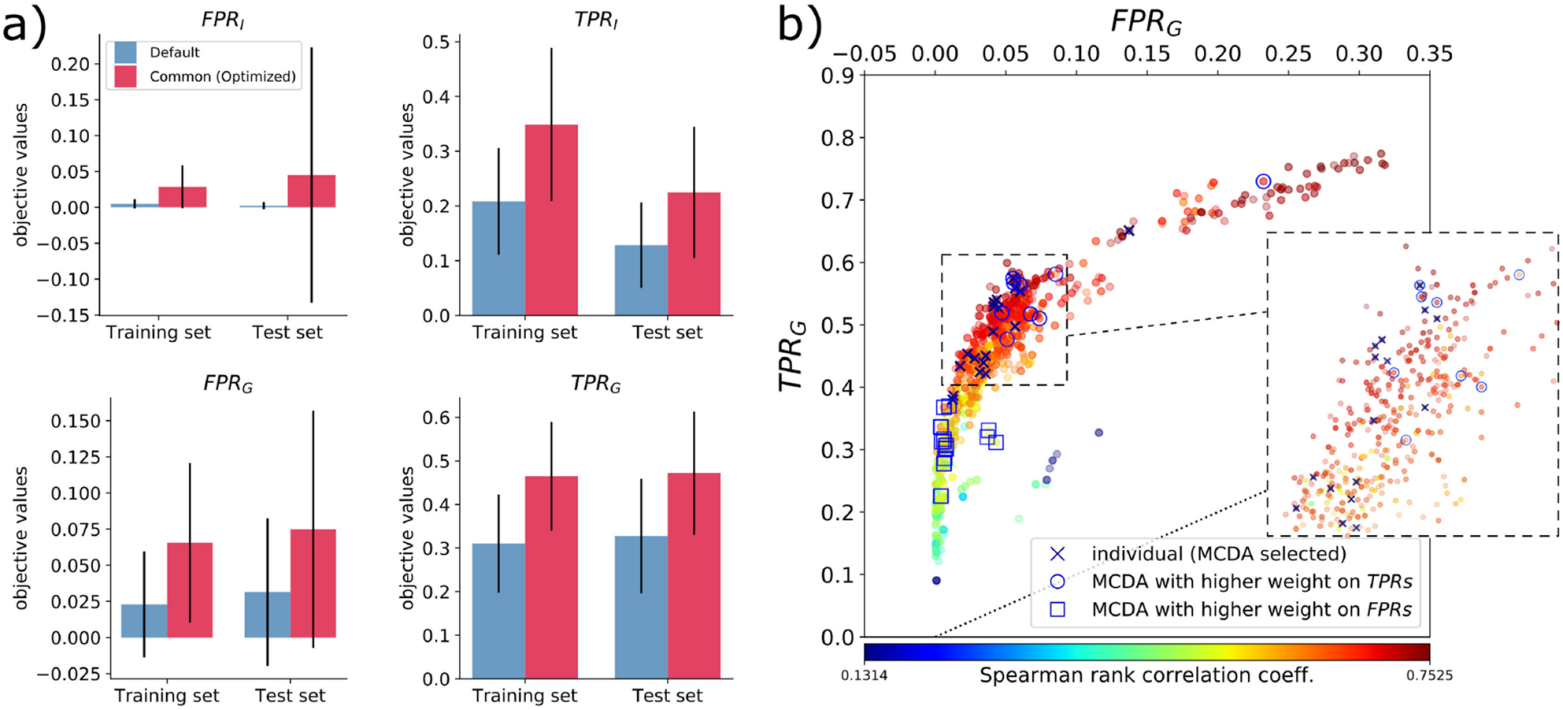
Performance on test data and comparison with training results for iFOD2. (**a**) Comparison of objective’s performance between training and test sets (average values from 5 runs for each brain) shows improvement of $TPR_I$ and $TPR_G$, and growth control of $FPR_I$ and $FPR_G$ for the optimized generic settings. $TPR_G$ reveals similar results for test and training sets, suggesting good generalization capabilities of optimized parameters for full-connectome estimation. (**b**) ROC space and Spearman’s rank correlation coefficient (color coded) for dMRI- versus neural tracer-based connectomes ($TPR_G$ versus $FPR_G$). Best individual solutions (from MCDA) are indicated by dark-blue x markers. Examples of differently weighted criteria during MCDA selection process are presented as blue circles and squares. The former case implements higher weights on *TPR*’s objectives, shifting solutions to better values of $TPR_G$, while the latter shifts to better values of $FPR_G$ by assigning higher weights to the *FPR*’s objectives.

Standard settings are validated by performing 5 fiber tracking runs on the training and test sets, averaging objective values for each set, and comparing with the corresponding default performance (Fig. 4a). $TPR_G$ improved notably from $0.32\pm 0.13$ to $0.472\pm 0.14$ (test set) and from $0.3\pm 0.11$ to $0.46\pm 0.12$ (training set). Both performances are similar to those of individual winners above, which suggests the robustness of optimized parameters in enhancement of wide-brain, region-level connections. $TPR_I$ advanced to better values; however, different performances are evident between test and training sets, possibly due to individual variability of the brains. $FPR_I$ and $FPR_G$ growth was controlled efficiently, with values close to 0. $FPR_G$ moved slightly from $0.03\pm 0.05$ to $0.07\pm 0.082$ (test set), and from $0.023\pm 0.037$ to $0.065\pm 0.05$ (training set). Low values of *FPR* along with a fixed fiber density demonstrate the efficiency of the framework in constraining the dominance of *FP*.

Best solutions were mapped onto the $TPR_G$ versus $FPR_G$ ROC curve (Fig. 4b, dark-blue x markers). The objectives, by MCDA decision criteria, were equally weighted in the solution selection process. An example of differently weighted criteria, in which *TPR* weights are defined slightly above other objectives, shifts winners to better values of $TPR_G$ (blue circles). On the other hand, weights on *FPR*’s shift winners to better values of $FPR_G$ (blue squares). Spearman’s rank correlation coefficients from $20\times 500$ connectome comparisons are color coded. Best solutions, on average, reached a correlation of $0.67 \pm 0.05$.

### Global fiber tracking with fiber-passage criteria

In the second experiment, we take the global fiber tracking algorithm^27^, which tracks long-range connections better than seed-based methods (DMFC-fiberCup at MICCAI’2009). We explore the major parameters: width $\sigma$, length *l*, weight *w*, chemPot *c* and connlike *L*^27^ (see Global tractography and parameter selection at “Methods” section).

#### Criteria for evaluation

Fitting can be quantified for axon trajectories at the voxel level or for projection targets at the brain-region level. An important issue in dMRI-based fiber tracking is the difficulty of tracking long connections, such as cross-hemisphere or sub-cortical connections. Accordingly, we consider the following four objective functions (Fig. 1b): (i) distance-weighted coverage, (ii) the true/false positive ratio, (iii) projection coincidence, and (iv) commissural passage, as explained below.

(i) **Distance-weighted coverage** $f_1=TPR^w_v=\frac{\sum _i^{N_{TP}}P_i}{\sum _i^{N_P}{P_i}}$. Here, $P_i=\frac{d_i}{max(d)} \times \frac{w_i}{max(w)}$ is a positive voxel in the 3D tracer image reconstruction that is weighted by voxel fluorescence intensity $w_i$ and the distance $d_i$ from the voxel to the center of the injection region. This objective is maximized and uses $d_i$ and $w_i$ to promote long-range connections, with voxels strongly connected to the injection region. $N_{TP}$ is the total number of true positive voxels found in the comparison, and $N_P$ the total number of positive voxels in the tracer data.
(ii) **True/false positive ratio** $f_2=\frac{TPR^w_v}{FPR_v+\epsilon }$. Here, $FPR_v$ is the false positive rate at the voxel-level, and $\epsilon$ is the tolerance term calculated empirically and given by $\epsilon =0.006\times \frac{\mu _P}{\mu _N}$, with $\mu _N$ equal to the average number of true negative *TN* voxels within individual whole-brain masks for the training data set, and $\mu _P$, similarly, the mean number of true positive *TP* voxels. $\mu _N$ is a large number. $\epsilon$ provides the minimum acceptable value of $FPR_v$, considering for example, that tractography results would be adequate, even if up to 0.6$\%$ of the *TP* are missed and counted as *FP*. Our optimization used $\epsilon =0.0013$. Maximization of this objective drives $TPR^w_v$ growth and maintains $FPR_v$ below a reasonable level, helping to constrain the dominance of *FP*^26^. We observed cases in which small increments of $FPR_v$ resulted in maximization of (ii); thus, we added (i) cost explicitly to adjust (ii) in the right direction.
(iii) **Projection coincidence** $f_3=r_{contra}$, the Spearman’s rank correlation coefficient between neural tracer and dMRI tractography-based connectome matrices for the contralateral-hemisphere of the brain. This objective function promotes accuracy of long cross-hemisphere projections. Global tractography was run twice with the same parameters, and results were averaged and mapped to the tracer-based connectome matrix of 20 injection regions $\times$ 104 targets parcellation. Both matrices were log-transformed and normalized.
(iv) **Commissural passage** $f_4=\frac{P_{out}}{V_{out}}$. While direction-insensitive dMRI fiber tracking should yield many “false positives” in reference to anterograde neural tracers, some estimated paths are impossible, such as those crossing hemispheres outside of commissural areas. This criterion uses a binary mask at the midline, covering voxels outside anatomical commissures, such as the corpus callosum and the cerebellum. $P_{out}$ is the number of voxels for fibers crossing the mid-line outside commissures, and $V_{out}$ is the total number of positive voxels of the mask. This objective is targeted for minimization, and supports the non-dominance of *FP*. $f_4$ is additionally evaluated as $f_4^*=\frac{P_{in}}{P_{in}+P_{out}}$, where $P_{in}$ counts the voxels of fibers passing through the anatomical commissures. $f_4^*$ provides the proportion of true anatomical reconstructions at the commissures and the optimization accuracy for the interconnection of the two sides of the brain.

#### Multi-objective optimization

We took the same MOO approach using NSGA-II^35^ as in the previous experiment (see Optimization at “Methods” section). The process optimizes several brains in parallel; however, because of computational demands for the global tracking algorithm, we added parallelization at the fitness function calculation and prepared the code for HPC clusters. In this way, we perform several global tracking runs simultaneously (see Code implementation at “Methods” section).

To verify the consistency and convergence of optimized parameters across subjects, we visualize the evolution of the five parameters and four objectives for all ten training samples (Supplementary Fig. S2b). Optimization started with parameters at their default values (dotted line) and widely explored values within the defined search ranges. Over generations, parameters for all brains converged to similar loci while improving the objectives. *width*, *weight* and *chemPot* converged to almost the same value (see late iterations), whereas due to brain heterogeneity, *length* and *connlike* followed different paths to achieve the best results. This serves as an indicator of parameter robustness for generalization.

We chose standard parameters (the generic setting) by considering trade-offs between objectives (see choice of standard parameters by MCDA below) and using the mean and standard deviation of the best-scoring parameters (shown by red dots and bars in Supplementary Fig. S2b and Table 1).

**Figure 5:**
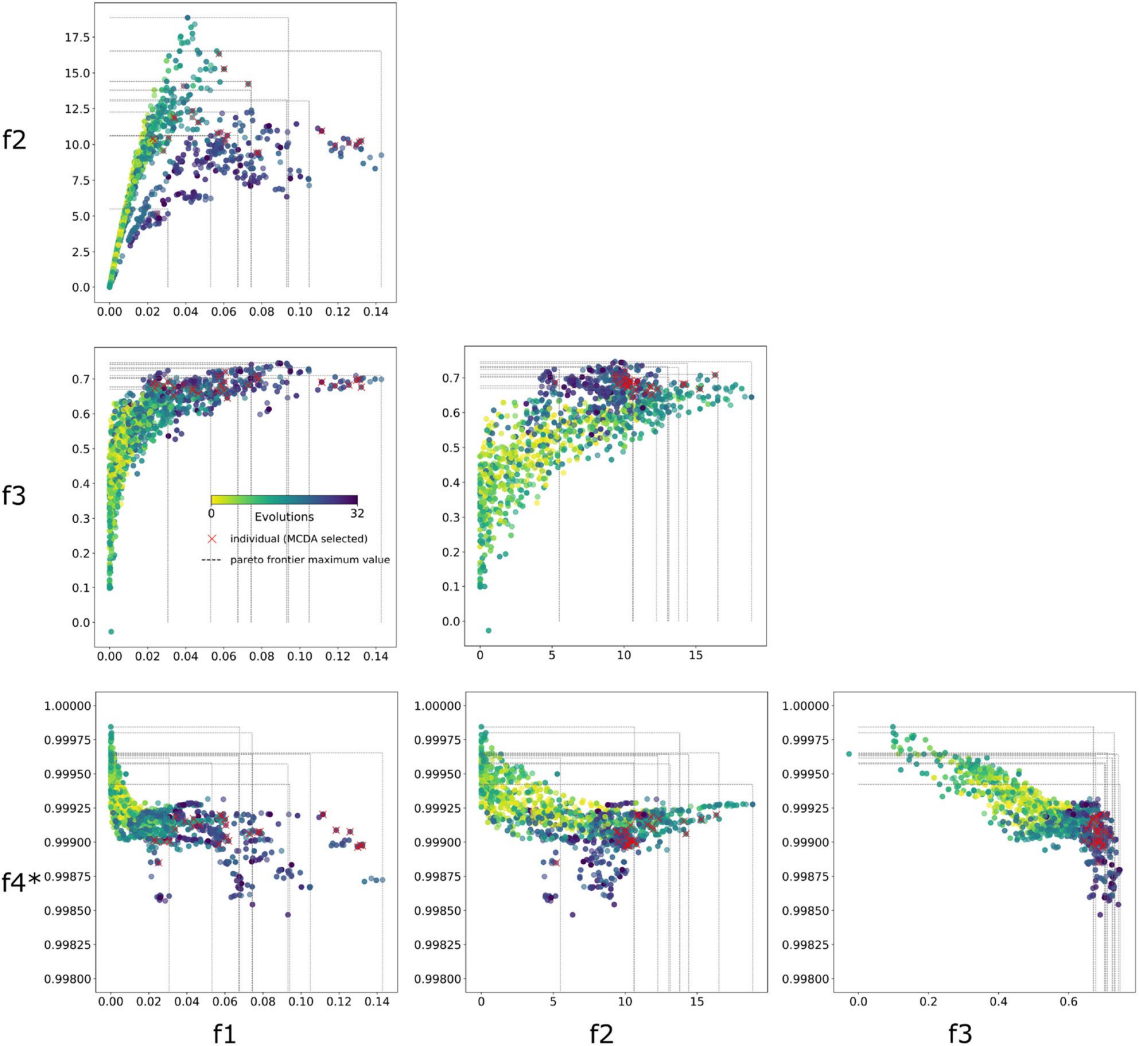
Objective function optimization for global tracking. Pair-wise visualization of the optimization of the four proposed objective functions: $f_1$: distance-weighted coverage, $f_2$: true/false positive rate, $f_3$: projection coincidence, and $f_4^*$: commissural passage. Our framework drives objectives toward the Pareto front in the upper-right direction. MCDA-based best objective trade-offs across brains are shown as red x markers. The standard setting is computed as their mean and standard deviation.

To evaluate optimization of multiple objectives, we visualize the pair-wise evolution of objectives (Fig. 5). Multiple Pareto frontiers were developed (1 per brain), which are most clearly seen in $f_1$ versus $f_2$ with dotted lines passing through the Pareto’s extremes (maximum value of *f*). This may be caused by subject individuality; however, systematic sharing of “champions” enabled the algorithm to achieve optimal parameters in a similar locus among brains.

Competing goals $f_1$, $f_2$, and $f_3$ were “pushed” by the optimization from the lower-left (default parameters) to the upper-right region (optimized parameters) as seen in $f_1$ versus $f_2$, $f_1$ versus $f_3$ and $f_2$ versus $f_3$. $f_4^*$ maintained the proportion of valid fibers connecting hemispheres, a critical condition when the number of fibers increased and the tractography became denser. $f_1$ versus $f_4^*$, $f_2$ versus $f_4^*$, and $f_3$ versus $f_4^*$ indicate that $99\%$ of the crossing fibers passed through valid commissural voxels.

Results of fiber tracking with and without parameter optimization are visualized by overlapping dMRI-based fiber-density maps (red) with neural tracer data (green) (Fig. 3b and Supplementary Fig. S1b). Default settings generate sparse coverage, characterized by a few short fibers connected to the injection region. In contrast, tractography with optimized parameters presents expanded overlap with tracer signals, demonstrating higher sensitivity. Longer fibers were connected not only to neighboring high-concentration neural tracer regions, but extended to cross-hemisphere areas and distant areas within the same hemisphere. The true/false positive ratio $f_2$ and the commissural passage $f_4$ allow control of the volatile growth of *FP*, while sensitivity and long-range connections are supported by the distance-weighted coverage $f_1$ and the projection coincidence $f_3$.

We monitored the number and mean length of fibers estimated by tractography in the course of optimization (Supplementary Fig. S2a). Both metrics increased from their default values of approximately 50, 000 fibers and 10 mm to optimized values of about 200, 000 fibers and 17 mm (see fiber length performance for a brain example at Supplementary Fig. S3). Higher fiber density helped to increase sensitivity in comparisons with tracer data, while longer fibers promote distant connections between source-target pairs. However, fiber density must be constrained to avoid unrealistic results, controlled in our framework by $f_2$ and $f_4$.

#### Choice of standard parameters by MCDA

We used MCDA to select the best trade-off solutions and the standard set of parameters as in the previous experiment.

Rated parameters were arranged in a matrix of $40 \times m$, where 40 is the arrangement of the 4 objectives $\times$ 10 brains and *m* is the number of parameter settings over the optimization. After averaging rates across *f*’s, the maximum scored parameters were selected as the winner(s) for the brain (Supplementary Fig. S4). Finally, the standard set of parameters is obtained using the mean and standard deviation of the winning parameters for the 10 brains. The result is shown in Table 1 along with the default parameters.

**Table 1:** Standard parameters for global tracking obtained by multi-objective optimization and MCDA over multiple marmoset brains.

Parameters generalization		
Parameters ($\theta$)	Optimized value (mean ± std)	Default value^27^
*width* ($\sigma$)	0.07 ± 0.005	0.1
*length* (*l*)	0.45 ± 0.043	0.3
*weight* (*w*)	0.054 ± 0.027	0.133
*chemPot* (*c*)	0.106 ± 0.032	0.2
*connlike* (*L*)	0.86 ± 0.23	0.5

#### Validation

To validate the effectiveness of optimized parameters above, we compared training and test datasets in terms of the proposed objectives, for default and optimized parameters. First, considering only the training set, we performed 5 global tractography runs for each default and optimized setting. In the latter case, each value is drawn from a normal distribution, with its mean and standard deviation as described in Table 1. Tractography results are averaged for each brain and shown along with the performance of MCDA selected winners, for comparison (Supplementary Fig. S5).

For individual winners and common standard parameters, on average, $f_1$ obtained values of $0.067\pm 0.036$ and $0.024\pm 0.012$, $f_2$ values of $11.24\pm 1.98$ and $7.38\pm 1.88$, $f_3$ $0.68\pm 0.016$ and $0.62\pm 0.06$, and $f_4^*$ 0.99 and 0.99, respectively. The standard parameters generalize well for improving cross-hemisphere projections ($f_3$) and commissural passage ($f_4^*$). For $f_1$ and $f_2$, although the standard parameters achieved lower scores than the winners, they outperformed the default settings. Compared to the results with default parameters, on average, $f_1$, $f_2$ and $f_3$ advanced from their low values ($0.003\pm 0.002$, $2.3\pm 1.4$ and $0.4\pm 0.05$, respectively) to considerably better, optimized values (as shown above), reaching a superior distance-weighted coverage $f_1$, while constraining false positives through $f_2$ and $f_4$. $f_4^*$ showed similar results for the three sets of parameters.

For the default case, coverage is low, and few fibers were generated, which leads to a high value of $f_4^*$. However, when $f_1$ increased by optimization, many more fibers were estimated. A high value of $f_4^*$ indicates a similar level of accuracy at the commissural passage.

**Figure 6:**
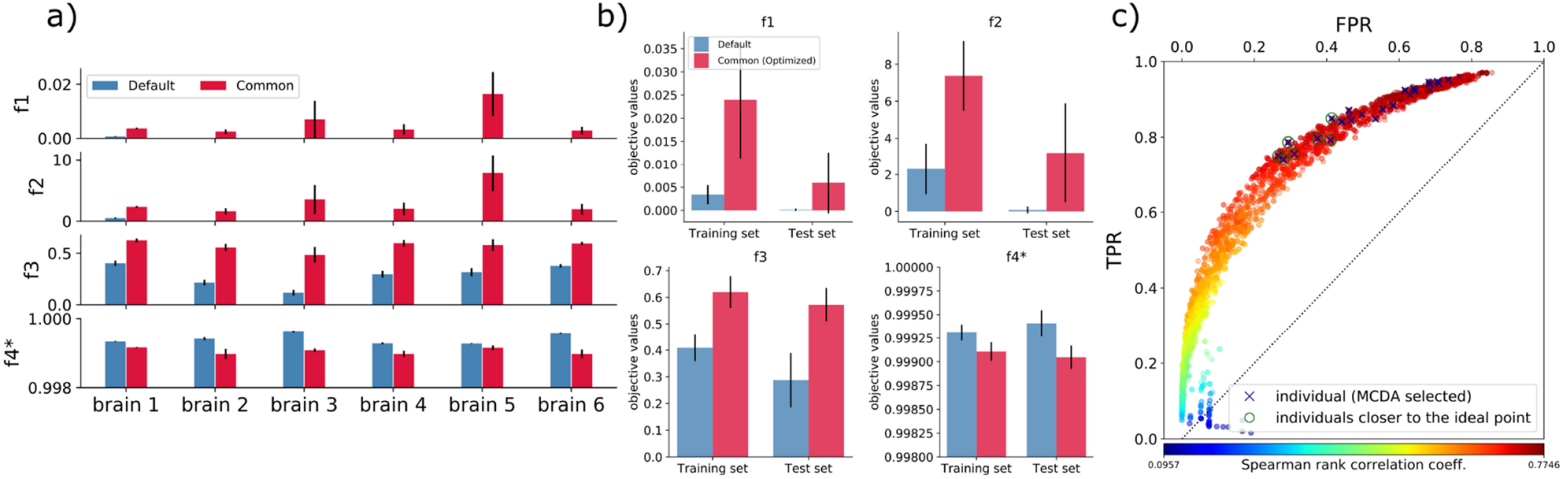
Performance on test data and region-level connectomes. (**a**) Objective function comparison (average values for 5 runs) for 6 additional marmosets shows improvement of $f_1$, $f_2$ and $f_3$, and consistency of $f_4^*$. (**b**) Performance comparison between training and test data sets for the default and optimized settings. $f_3$ is the most improved objective; however, improvement of $f_1$ and $f_2$ contributed to better results, as well as $f_4^*$ consistency for denser tractograms. (**c**) Spearman’s rank correlation coefficient (color coded) mapped onto *TPR*-*FPR* space, from comparisons of 20$\times$500 neural tracer- and tractography-based matrices, for the entire optimization process. Optimized tractography results (dark blue x’s) closer to the ideal ROC coordinate (green circles) show high correlation.

Generalization capability of optimized parameters is also evaluated on 6 unseen marmoset brains (test set, Fig. 6a). We ran tractography 5 times using default parameters and standard optimized parameters. Results show improvement for $f_1$, $f_2$ and $f_3$, for all brains. $f_1$ improved on average from $0.0001\pm 0.0002$ to $0.006\pm 0.006$, $f_2$ from $0.08\pm 0.18$ to $3.2\pm 2.7$ and $f_3$ from $0.28\pm 0.1$ to $0.573\pm 0.06$. As expected, $f_4^*$ showed similar results of about 0.99.

Figure 6b summarizes the averaged performance for training and test data sets, showing similar results. The objective $f_3$ exposes better generalization performance.

Optimized parameters improved results in terms of the desired objectives for both cases, validating the proposed standard parameter settings. The improvements are clearly recognized in Supplementary Fig. S6 for a brain sample, which visualizes in high-resolution the ground-truth neuronal tracer signal (green) 3D reconstruction, and the global tracking fibers (red) in contact with the injection region, as density maps. Optimization improves fiber-density map matching with the neuronal tracer. Standard parameters perform similarly with decreased density results.

$f_1$ reports small values as a result of thousands of neural tracer voxels averaging the coincidences with voxels covered by fibers, and the mapping of fibers to a high-resolution space (standard brain). We evaluated the strength-weighted coverage $f_1^*=\frac{\sum _i^{N_{TP}} w_i}{\sum _i^{N_P} w_i}$ of axonal tracts at the voxel-level for the training set (see Supplementary Fig. S7) over the generated parameter settings. The coverage improved on average from 0.9% (default) to 15% (MCDA selected winners).

Finally, we evaluated region-level connectome matrices estimated by dMRI-based tractography in reference to the Brain/MINDS marmoset connectome data over the course of the optimization. Tractography-based matrices were mapped to the $20\times 500$ structure, as with group objectives from the 1st experiment. We calculated Spearman’s correlation, TPR and FPR (Fig. 6c). The best optimization results (blue x’s) substantially overlap settings close to the ideal ROC point (0.0, 1.0) (green circles), and reported on average: $FPR=0.33$, $TPR=0.78$, distance to the ideal point $d=0.163$, and correlation coefficient $r=0.724$. Qualitatively, improvements are recognized by matrix visualization, using coarse-grained parcellation for a brain sample (Supplementary Fig. S8). Compared to the sparse connections using default parameters (bottom matrix), tractography using optimized parameters (center matrix) revealed denser and longer connections, enhancing connectivity to projection areas in the right-hemisphere (left half of the matrices) from their origins in the left hemisphere. Optimized dMRI-based tractography can complement the sparse structural network obtained from tracer injections (top matrix).

### Comparison of tracking algorithms and objectives

For both, iFOD2 and global tracking algorithms, optimization increased spatial coverage and fiber length (Fig. 3, Supplementary Fig. S1), with better performance for the global tracking case. Fiber length improved on average 4 mm (8–12 mm) for iFOD2 and 7 mm (10–17 mm) for global tracking. Comparison between neural tracer- and dMRI-based connectomes (Fig. 4b versus Fig. 6c) exposes lower values of $TPR_G=0.5$ and $FPR_G=0.04$ for iFOD2 against global tracking performance of about $TPR_G=0.78$ and $FPR_G=0.33$ .

**Figure 7:**
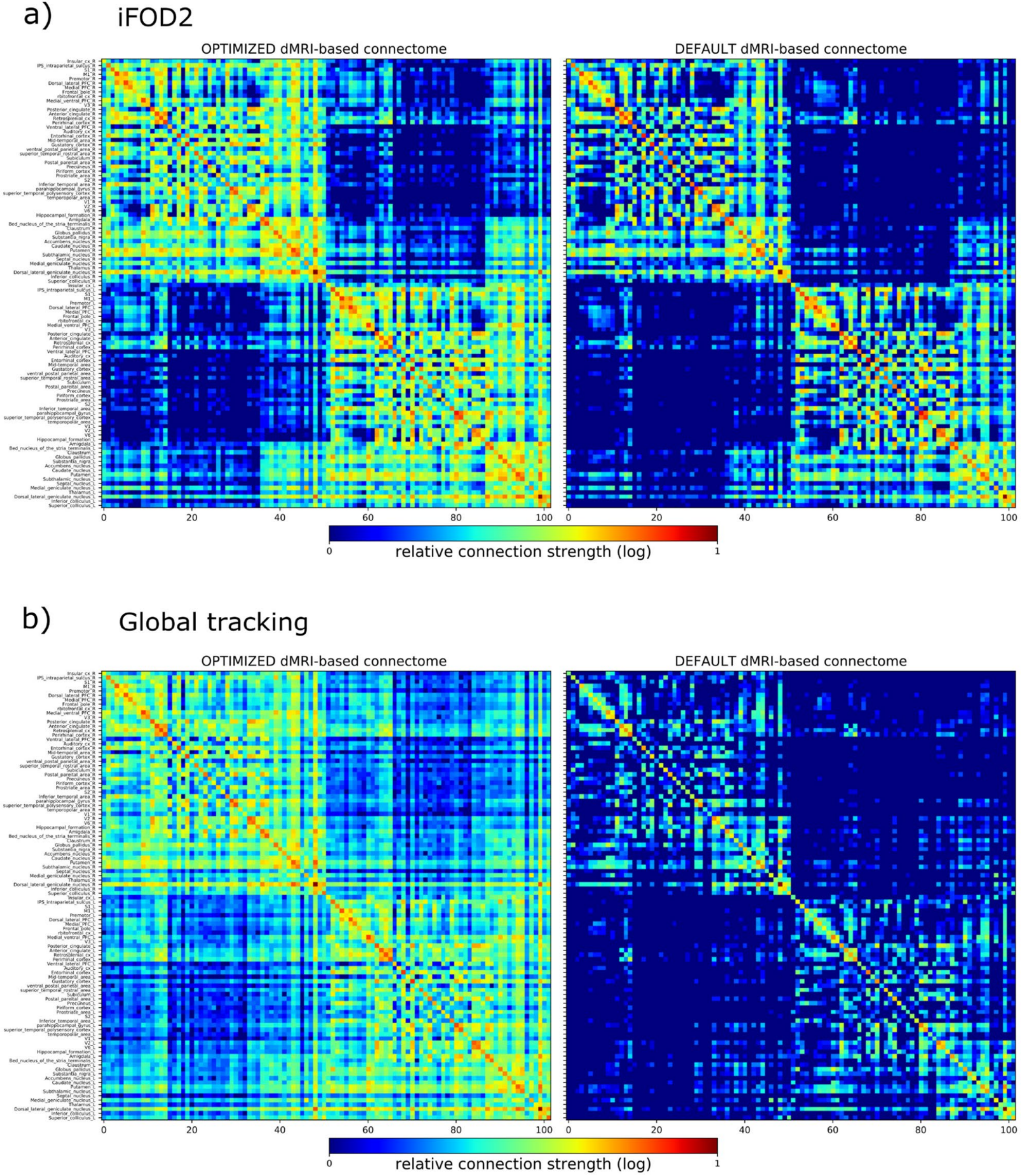
Whole-brain dMRI-based optimized and default connectomes. Square dMRI-based matrix comparison for one brain-subject example, using optimized (left) and default (right) parameters for (**a**) iFOD2 and (**b**) global tracking algorithms.

The main causes of these results are: global tracking implemented “tolerance” for *FP*, while iFOD2 optimized objectives to explicitly control *FP*. Tolerance is relevant because dMRI-based tractography finds both incoming and outgoing fibers to and from an ROI, compared to an anterograde tracer-based connectome of only outgoing fibers. Some “false” positives are reasonable. In the global tracking experiment, a tolerance term $\epsilon$ is specially implemented by $f_2$; however, constraints on *FP* at voxel-level in comparisons with neural-tracer 3D reconstructions ($f_2$) and at the commissural passage ($f_4$), provided additional *FP* tolerance for fibers estimated outside the boundaries implicitly defined by the objectives, namely unconnected fibers from the injection region, fibers outside the coverage of tracer references, and fibers not crossing commissures. On the other hand, the iFOD2 case minimized group and individual *FP*, at region-level connectomes built from tractograms with a fixed fiber density.

Nevertheless, Spearman’s rank correlation coefficients (average of the best solutions) for both cases reveal similarities: $r=0.67$ (iFOD2) and $r=0.724$ (global tracking).

Consequently, connectivity is enhanced, not only from/to injected regions, but brain-wide (Figs. 3, 7), showing richer connection estimates for optimized cases. This demonstrates that better connectomes can be achieved by applying our framework, independently of changes in fiber density (see the fixed density case Fig. 7a). This suggests that despite using fractional references from tracer injections at the left prefrontal cortex, whole-brain connectivity can be improved.

Results of the two experiments demonstrate the general applicability of our framework to different fiber-tracking algorithms and evaluation criteria, and confirm the importance of objective design for improving fiber tracking (see “Discussion” section).

## Discussion

We optimized and validated parameters of fiber tracking algorithms^27,33^ by exploiting fluorescent tracer and dMRI data from the same marmoset brains in the Brain/MINDS project^30^.

**Figure 8:**
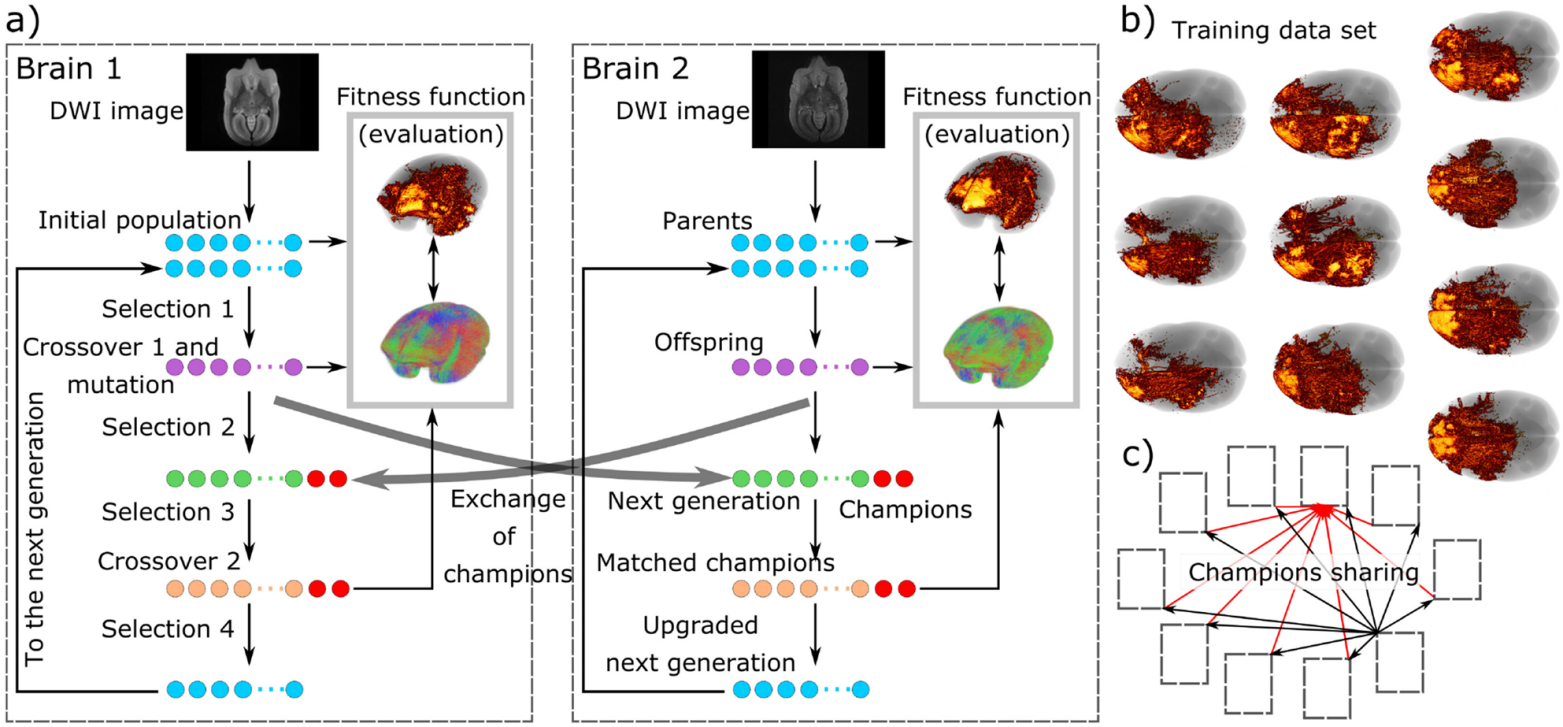
Multi-objective optimization (MOO) process. (**a**) From the initial population of parameters (parents, light blue dots), fitness values are obtained. Tournament selection (selection 1) creates offspring (purple dots). Crossover and mutation are performed on offspring and fitness values are calculated. From the combined set of parents and offspring, selection of the best elements (selection 2) creates the next generation (green dots). The best elements are shared between brain optimization processes (red dots). Most dominant elements (selection 3) are taken from the next generation and mixed with champions via a crossover operation. After obtaining objective values for matched elements and original champions, the next generation is upgraded by selecting the best elements from the joint set “next generation + original champions + matched champions” and sent as a parent for the next iteration. (**b**) One MOO runs for each brain of the training data set, (**c**) sharing the *i*-generation champion with all MOO-processes (black arrows) and receiving external champions as well (red arrows). Figure created using The MRtrix viewer 3.0.1 (https://www.mrtrix.org/) and Inkscape 1.0beta2 (https://inkscape.org/). Image datasets are part of the Brain/MINDS project (see Data availability section).

To address competing goals of sensitivity and specificity for multiple brains, we took a parallel, multi-objective optimization framework. Optimization was based on an NSGA-II evolutionary approach and implemented champion parameter sharing across brains to promote parameter generalization while maximizing objectives (Fig. 8).

For the iFOD2 algorithm, four objective functions (Fig. 1a) were used for region-level assessments: two group objectives ($TPR_G$ and $FPR_G$ rates, for comparisons with Brain/MINDS tracer connectome data from 20 marmosets), and two individual objectives ($TPR_I$ and $FPR_I$ rates, for comparisons with individual tracer injection data). Optimization constrained *FP* efficiently to values below 4% on average (Fig. 2), driving the correlation with Brain/MINDS connectome areas to 67% (Fig. 4b), and resulted in important increases of *TP* (Fig. 4a). With respect to default performance, $TPR_G$ improved from 30% to 50% (Fig. 2), and the average fiber length from 8 mm to 12 mm (Fig. 3a, Supplementary Fig. S1a). Improvements were independent of growth/loss of fiber density. Instead, they relied entirely on better parameters.

For the global tracking algorithm, we developed four objective functions (Fig. 1b); two voxel-level objectives ($f_1$: distance-weighted coverage, $f_2$: true/false positive ratio), a region-level objective ($f_3$: projection coincidence), and an anatomical constraint ($f_4$: commissural passage). During optimization, while constraining impossible fibers at the commissural passage and controlling the growth of false positives, our framework improved dMRI-based fiber tracking performance with respect to default values: average fiber length from 10 to 17 mm (Fig. 3b, Supplementary Fig. S1b,  S2 and S3), voxel-wise coverage of axonal tracts from 0.9% to 15% (Supplementary Fig. S7), and correlation of target areas from 40% to 68% (Supplementary Fig. S5).

Originally, we started this effort by optimizing a single objective function, such as $C^2 = FPR_v^2+(1-\frac{\sum _i^{N_{TP}}d_i}{\sum _i^{N_P}d_i})^2$, where the second term is the normalized sum of distances from *TP* voxels to the center of mass of the injection region, similar to $f_1$, but using only $d_i$ as a weighting factor. However, results for the combined single-objective function by the co-variance matrix-adaptation evolution strategy (CMA-ES)^36,37^ were unsatisfactory, with a huge density of fibers, dominance of false positives, and many fibers crossing hemispheres outside the commissures.

An important feature of our work is that comparisons of dMRI and reference data are performed in parallel for multiple brains, which can account for individual variability. From the multiple Pareto-optimal solution for multiple brains, we used an MCDA method to select a standard set of parameters (see Multiple criteria decision analysis for standard parameters, and Table 1). Excluding brain samples used for optimization from the test set, we verified that the standard parameters substantially improve fiber tracking performance compared to the default parameters (Figs. 4,  6,  7 and Supplementary  S8). Standard parameters generalized better on objectives evaluating connectome similarities ($TPR_G$, Fig. 4) and correlations ($f_3$, Fig. 6a,b), whereas the effect from individual subject variability is noticed in objectives measuring local features.

Improvements were similar for both experiments, but more prominent for the global tracking algorithm with more elaborated objectives and tolerance for *FP*. However, the 1st experiment verified improvements on a widely used tracking algorithm (MRtrix3), with simpler objective functions and lower computational requirements (see Code implementation at “Methods” section).

Results on unseen subjects demonstrate the generalizability of the standard parameters to marmoset. Although both experiments used reference data from 20 tracer injections at the prefrontal cortex, improved tracking was not limited to that area, but to the whole-brain, as illustrated in extended fibers in Fig. 3 and brain-wide region-to-region connectomes in Fig. 7. The Brain/MINDS marmoset connectivity map is an on-going effort. New reference data are expected in the short term; thus, our framework will re-run optimizations on complete data sets, setting the standard parameters reported here as initial conditions. In addition, an important follow-up work will be to verify whether the same solution applies to diseased animals, making new comparisons between dMRI and tracer data of those marmoset subjects.

Our optimization and validation framework can be flexibly applied to different tracking algorithms and objective functions, as demonstrated in the two reported experiments, as well as to different species. Complete tracer data sets exist for mice^38^ and macaques^39^, although having similar tracer data from human subjects would be difficult, our framework allows integration of multiple biological constraints^40^. Applying the method to other species will be important, not only for improving current results, but for verifying consistency/scaling of optimal parameters across species. The implementation code is available to the scientific community for improving accuracy and reliability of dMRI-based fiber tracking.

The framework allows assimilation of additional data as references. Recently, Zhang et al.^41^ proposed optimization of dMRI-based fiber tracking using the region-level coincidence with neural tracer data in the CoCoMac database^39^ and matching of fiber orientations with myelin staining data from a single macaque brain^42^. They took the average of Youden’s index (the sum of sensitivity and specificity)^43^ for connected regions and the coincidence index of fiber orientation as the criterion, and performed a grid search in a two-dimensional parameter space of a fiber tracking algorithm^44^.

Other possible references include molecular cues to targets^45^ and connectivity reported by electrophysiological experiments^46^. Multiple references are desirable, and the framework manages them in a data-driven manner. We think that more comparisons are better, despite low dMRI resolution and lack of directionality. Comparisons are beneficial in a wider sense from potentially improving cross fiber issues to clarifying the limitations of fiber tracking.

How to define the best objective functions from the available data, especially when the data sources are not strictly the ground-truth, but an approximation, poses new challenges. Our optimization defined equally weighted objectives to mitigate well-known issues of dMRI fiber tracking, but differently weighted objectives may work better. Objective functions can have one of two roles: “promoter” functions that maximize mapping between reference and estimated data and “constrainer” functions that minimize assumed incorrect data mapping.

A suitable definition of objectives will play an important role in avoiding over-promoted or under-penalized results. Incorporation of hybrid objectives, such as the True/False positive ratio, may suffice to mitigate unbalanced optimization.

Other important factors in choosing objective functions include whether to take global features, such as wide-brain connectome similarities or local features like axon trajectory mapping, at voxel-level.

Objective functions designed on top of noisy and partial observations of the ground-truth should allow tolerance for “false positives”, as in the case of incoming fibers to the injection region for anterograde neural tracer data. We designed the multi-objective framework to equally improve important objectives while providing tolerance, considering that cohesion of optimized objectives in trade-off solutions leads to constraint of authentic undesirable fiber tracking estimations.

In that context, an additional challenge is how to choose the best solution from a multi-dimensional Pareto front. We took a multi-criterion decision analysis (MCDA) that implements criteria weighting and scoring. MCDA is useful when some objectives need to gain more than others due their relative importance, unbalanced condition or deficient objective set-up.

## Conclusion

We proposed a flexible framework that improves dMRI-based fiber tracking by multi-objective optimization using neural tracer data as a reference. The framework runs with data from multiple brains cooperatively and in parallel. It was tested on different tractography algorithms, parameters, and objectives, and showed improvements in terms of defined objectives and other criteria for training and test data sets.

Multiple objective functions were designed to address critical issues in dMRI tractography. For iFOD2 algorithm, the parallel optimization process constrained successfully false positives, while increasing sensitivity. For global tracking algorithm, it promoted sensitivity, strong, long-range connections and high correlation with contralateral projection areas, while controlling unrealistic fibers at the commissural passage and false positives in comparison with neural tracer.

These results indicate the importance of optimization and validation of dMRI-based fiber tracking algorithms and also raise concerns about connectome studies that lack validation of fiber tracking algorithms.

There is a real opportunity to exploit multi-modal data being generated by multiple global brain projects to establish reliable methods for inferring brain structures, functions, and their relationships.

Our work provides the framework to implement it.

## Methods

### Statement on the use of experimental animals

Marmosets were not directly used in the present work. Imaging data were obtained in a separate collaborative study, and will be made available upon publication of the corresponding study.

Although there was no direct use of experimental animals, we want to emphasize that fluorescent neural tracer experiments and diffusion-weighted magnetic resonance imaging in Brain/MINDS were conducted with approval of the Animal Experiment Committee of RIKEN, in compliance with all required regulatory and ethical guidelines.

### Optimization

The non-dominated sorting genetic algorithm II (NSGA-II)^35^ is arranged for parallel optimization of the training set (Fig. 8).

**1st experiment initial settings** Parameters $\theta$=[angle, cutoff, minlength] are initialized by their default values $\mu _{\theta }=[45,0.1,2.0]$, while exploration ranges are settled heuristically with lower [10, 0.01, 1.0] and upper [90, 1.0, 18.0] bounds, respectively. A population *M* of size 8 is drawn from random uniform distributions with mean $\mu _{\theta }$ and standard deviation $\sigma _{\theta }=0.01$, except for $\sigma _{\text{ cutoff }}=0.001$. Each element $M_i$ of *M*, called an *individual*, is an array of length 3, corresponding to the parameters to optimize $\theta$.

**2nd experiment initial settings** Parameters $\theta$ = [width $\sigma$, length *l*, weight *w*, chemPot *c*, connlike *L*]^27^ (see Global tractography and parameter selection at “Methods” section) are initialized to their default values $\mu _{\theta }=[0.1,0.3,0.133,0.2,0.5]$^27^, and the exploration is defined within heuristically determined lower [0.01, 0.24, 0.01, 0.05, 0.5] and upper [0.15, 0.65, 0.22, 0.6, 6.0] bounds. A population *M* of size 8 is drawn from random uniform distributions with mean $\mu _{\theta }$ and standard deviation $\sigma _{\theta }=0.01$ except for $\sigma _{weight}=0.001$. Each individual $M_i$ is an array of length 5.

**Generational process** Fitness values $f(M_i)$’s of the initial population *M* are calculated and the generational NSGA-II^35^-based process begins. Depending on fitness values, tournament, dominance-based selection between 2 individuals $M_i$ is performed. If the $f(M_i)$’s pair does not inter-dominate, selection is accomplished by evaluating the crowding distance^35^. With repetition, the tournament selects 8 offspring. We choose to invalidate the fitness of the offspring and perform crossover and mutation directly. Crossover picks individuals at even positions of the offspring array and pairs them with individuals in odd positions. Crossover uses simulated binary crossover^47^, which is applied to each pair with probability $cxp=0.2$ of matching two individuals. Mutation is applied to all individuals among the offspring using a polynomial approach^47^. Offspring fitness values are calculated. Then, from the combined set of parents and offspring, the next generation of 8 elements is selected based on fitness values and spread^35^. In addition, the best individual is selected from the combined set as the local “champion.” Champions are shared among brains to promote convergence of parameters in a similar locus. A process barrier is used as a synchronization step to allow $n=10$ training brains to receive $(n-1)=9$ champions. Once all champions are shared, the process barrier is set to “OFF” and the process continues. From the next generation set, the 3 dominant individuals are selected by tournament^35^ and added to the champion set. Crossover with $cxp=1$ is applied to the extended champion set by matching even- with odd-positioned individuals, as in the preceding matching step. We process fitness values for the original and matched champions and a final selection of the best 8 individuals from the total set “next generation + original champions + matched champions” is used to upgrade the next generation set *M*. From *M*, in like manner, offspring are selected and the process continues for another generation.

**1st experiment adjustments** From the 8th evolution, bounds were constrained to [25, 0.05, 1.0] (lower) and [55, 0.5, 10.0] (upper) to accelerate the optimization process.

**2nd experiment adjustments** The process explored several parameter values widely, and after several iterations, it gradually exposed a bifurcation of the inspection. Most of the parameters roughly followed an exploration path on each side of the default value. In order to decide which path leads to advancement of objectives, we compared objective values (Supplementary Fig. S2b). The comparison helps to constrain exploration by reducing searching intervals toward better values and less computation time, speeding-up optimization. The new exploration lower [0.01, 0.32, 0.01, 0.01, 0.1, ] and upper [0.10, 0.65, 0.13, 0.22, 3.0] bounds, achieved parameter stability after approximately the 20th iteration.

Both optimizations ran for 10 brains in parallel (training set) and stopped when slight changes in parameters produced almost no change in objective values, reaching $E=32$ generations. Because optimization calculates fitness values twice for each generation (3 times for the initial one), the total number of iterations for each brain was $E^*=32\times 2+1$.

### Code implementation

The method reported here was implemented on a cluster HPC computer for global tracking algorithms. It processes several brains in parallel while sharing champion settings over the generations. Separate jobs are generated (1 job per brain) and synchronized for sharing. Jobs keep running evolutionary processes and were tested on a single core with low memory.

An additional parallelization of the fitness function was added due to the computational challenges of global tracking. It allows several runs of global tracking at the same time. A single global tracking run takes from 1 to 3 h for the initial generations. Fiber density and length increase gradually while improving the parameters. Then, every run becomes computationally expensive. For each fitness function calculation, the synchronized jobs dispatch 8 “heavy” jobs (1 job per individual parameter setting). A “heavy” job uses more than 1 core and requires higher memory for reading data sources (masks, neural tracer reconstructions, dMRI, atlas, injection regions), performing global tracking *n* times, calculating objective functions, and recording results (jobs information, parameters, tractograms, density maps, champions, connection matrices, objective values) in a folder-organized structure.

By this method, our framework parallelization is implemented at the level of individual brains and global tracking runs. The whole optimization process took around 4$\sim$5 weeks.

Additionally, an alternative portable implementation is made available for desktop PC’s, targeting commonly used tractography approaches that do not require important computing resources. This version exploits mpi4py^48^ to run parallel evolutionary processes, one per brain, while the fitness function runs sequentially within each process. Champion sharing and process synchronization are implemented as well. The iFOD2 algorithm optimization used this version of the code, obtaining results in less than 50% of the HPC implementation running time. Beside the fiber tracking algorithm, the complexity of objective functions and the number of fibers to generate may change the performance.

### Fluorescent neural tracer data

Segmented neural tracer 3D images (Figs. 3,  8b, Supplementary Fig. S1 and  S6a) were generated by marmonet^32^. Marmonet is the Brain/MINDS AI-driven pipeline for automated segmentation of tracer signals. It incorporates state-of-the-art machine learning techniques based on artificial convolutional neural networks^49^ and robust image registration. Raw images show the fluorescent signal of an anterograde tracer, a protein-based virus that tracks axons from injection region cells to their point of termination. Images are taken using two-photon microscopes, TissueCyte 1000 or TissueCyte 1100. Initially, they show several patterns, shapes, contrasts, and intensities. After marmonet pre-processing, image stitching, and segmentation, high-contrast results of the injection region and its center, corresponding cell bodies, and axon tracers are obtained. Segmentation results include voxel-intensity weighting from the raw tracer signal. All processed images are mapped from their $1.39 \times 1.34 \times 50\, \upmu \mathrm{m}^3$ resolution to the Brain/MINDS reference image space of $100 \times 100 \times 200 \, \upmu \mathrm{m}^3$ resolution. Tracer injection regions and their centers as 3D reconstructions were used in our optimization as well.

Despite differences between neural tracer and dMRI tractography, important voxel-level features from the 3D tracer segmentation images were exploited by the framework to improve fiber tracking results. We considered voxel intensity and its distance to the injection region as important features to promote strong, long-range connections in dMRI-based tractography. Thus, we assumed both features as common characteristics.

### Diffusion MRI

dMRI data were generated by ex-vivo marmoset experiments. Marmosets were perfusion-fixed (Table 2) and cranial brains were extracted. Brains were immersed in PFA reagent for 2–3 days, which was then replaced with PBS reagent. MRI imaging was performed on brains immersed in fluorinert liquid. A 9.4-Tesla small-animal MR scanner was used, controlled with a Bruker Paravision 6.0.1. The solenoid coil had an inner diameter of 28 mm. Diffusion imaging was accomplished using a spin-echo diffusion-weighted, echo-planar imaging sequence with repetition time $TR= 4000\,\mathrm{ms}$, echo time $TE = 21.8\,\mathrm{ms}$, and *b*-value $= 5000\,\mathrm{s/mm}^2$. The acquisition matrix was $190 \times 190 \times 105$ over a $38 \times 38 \times 21\,\mathrm{mm}^3$ field-of-view (FOV), resulting in a native isotropic image resolution of $200\, \upmu \mathrm{m}$. The diffusion sampling protocol included 128 unique diffusion directions and 2 non-diffusion-weighted (b0) measurements (the first b0 image was removed because it usually contains noise). Total acquisition time was 2 h 40 min per sample.

**Table 2:** Characteristics of marmoset brains used in this study. The same brains were handled for tracer injections and dMRI imaging.

Brains subjects used for optimization and validation			
Brain id	Gender	Fixed period (h)	Age (years until the day of sacrifice)
**Training data set**			
$R01\_0070\_CM1180F$	*F*	80	7
$R01\_0029\_CM696F$	*F*	48	6
$R01\_0072\_CM1176F$	*F*	45	11
$R01\_0030\_CM690F$	*F*	48	6
$R01\_0078\_CM1347F$	*F*	95	9
$R01\_0054\_CM1060F$	*F*	60	3
$R01\_0071\_CM1178F$	*F*	143	8
$R01\_0034\_CM521F$	*F*	48	3
$R01\_0039\_CM703F$	*F*	48	6
$R01\_0033\_CM694F$	*F*	48	6
**Test data set**			
$R01\_0026\_CM692F$	*F*	48	6
$R01\_0043\_CM628F$	*F*	52	4
$R01\_0040\_CM710M$	*M*	72	6
$R01\_0053\_CM1061F$	*F*	58	8
$R01\_0048\_CM1011F$	*F*	60	3
$R01\_0046\_CM1023M$	*M*	60	3

### Pre-processing

dMRI data, bvec and bval files, and individual whole-brain masks were acquired from the Brain/MINDS dMRI-pipeline. dMRI was de-noised using MRtrix3^34^ in 3 steps. First we applied *dwidenoise*, which exploits data redundancy in the PCA domain using random matrix theory^50,51^; secondly *mrdegibbs* removed Gibbs ringing artifacts by local subvoxel-shifts^52^. Finally, a mask filter was applied to the whole-brain mask, eroding 2 voxels to remove noise at the boundaries and to constrain abnormal fiber growth during fiber tracking. Injection region masks were dilated 2 voxels to improve detection of fibers contacting them, as support against potential bias in the registration and injection region detection. For registration tasks we used b0 images and advanced normalization tools ANTs^53^.

### Density maps

Evolutionary optimization requires comparison of fiber-density maps in standard brain space against neural tracer data (Fig. 1b). A fiber-density map is built for each individual (a particular parameter setting) using MRtrix3 commands. First, duplicated fiber tracking results are transferred from dMRI space to standard brain space by normalization mapping (*tcknormalise* or *tcktransform*). In the latter space, tractograms are intersected with the corresponding tracer injection region using *tckedit*. The resulting subset of fibers, as well as the complete tractogram, are converted to density maps by *tckmap* and averaged over the duplicated tractography runs. The density map corresponding to the subset of fibers is used for computation of $f_1$ and $f_2$. Similarly, $f_4^*$ is measured by the intersection of the commissural mask with the density map of the complete tractogram.

### Voxel weighting

Each voxel of $TPR^w_v$ ($f_1$ and $f_2$) is weighted with 2 factors obtained from neural tracer data, the distance $d_i$ and intensity $w_i$ (Fig. 1b). The center of the injection region contains few voxels. Refinement to a unique voxel is performed by summing all x, y, and z-coordinates and dividing each sum by the corresponding number of voxels, giving a unique 3D position. The updated center is used to calculate distances $d_i$ from all *TP* voxels to the injection center. Distances $d_i$ are normalized by the maximum observed distance. Neural tracer 3D images provide voxel intensities $w_i$, which are associated with connection strengths from the injection region. Similarly, $w_i$ are normalized by the maximum observed intensity.

### Global tractography and parameter selection

For the second experiment, dMRI-based tractography was performed using a global tracking algorithm^27^. This method provides the whole-brain connectivity configuration that optimally fits the acquired data^27–29^. The optimization applied is such that each particle (also called a segment) tries to mimic the source data, promoting its closeness to the measurement in anisotropic areas (e.g., the white matter), and inferring information in ambiguous isotropic areas (e.g., gray matter) by neighboring anisotropic areas. We selected this algorithm due to its documented reliability in terms of position, tangent directions, and curvature of reconstructed fibers with a phantom dataset at the DMFC-fiberCup at MICCAI’2009. However, it requires optimization for specific anatomy or species.

Global tracking does not use pre-defined seed(s), requiring no human intervention. Fibers are built with small line segments that form chains during tractographic optimization, and their number and orientation are adjusted to match data obtained from high angular resolution diffusion imaging (HARDI). From the set of segments and their connections, a predicted MR-signal is computed. Connection behavior between segments is controlled by internal energy from two parameters selected as relevant to our optimization: *length* *l* is the fiber segment length, and *connlike* *L* is the likeliness that two segments link together (also known as connection potential). External energy measures the difference between the current and predicted diffusion-weighted HARDI signals. From the external energy we designated as important parameters: the *weight* *w* contribution, and the *width* $\sigma$ of the prototype-signal of each segment. In addition, two more parameters were considered: the *chemPot*2 *c* (cost of adding a particle) and *chemPot*1 (similar to chemPot2, also known as the particle potential, which regulates the number and distribution of particles).

To test the significance of the selected parameters, we pre-evaluated them by running global tracking on 3 brains and assessing the fiber number and length variability caused by a single parameter change, while keeping others fixed at their default values (Supplementary Fig. S9). *Weight*, *width*, *length* and *connlike* produced changes in fiber density and length. However, changes of *chemPot*2 and *chemPot*1 values, produced almost no effect on fiber density and length, practically unnoticeable in the latter case. Therefore, we selected the first 4 parameters and *chemPot*2 (renamed as *chemPot*) for optimization.

## Supplementary information

Supplementary material 1.

## Data Availability

Optimization process code is publicly available on github (https://github.com/oist/gt_moo/). It can be adapted to other fiber tracking algorithms, data sources, and objective functions. The global tracking algorithm is available at https://www.uniklinik-freiburg.de/mr-en/research-groups/diffperf/fibertools.html. Datasets (neural tracer, dMRI, standard brain, atlas, neural tracer connectome, masks) will be made available as part of the Brain/MINDS project in the near future (data portal site: https://www.brainminds.riken.jp). All other data presented in this study are available from the corresponding author upon request.
