# Supplementary material for: Optimization and validation of diffusion MRI-based fiber tracking with neural tracer data as a reference
Source: Sci Rep. 2020 Dec 18;10:21285. doi: 10.1038/s41598-020-78284-4 (PMC7749185; doi:10.1038/s41598-020-78284-4)
Supplement: Supplementary file 1 — Supplementary material 1. [file 41598_2020_78284_MOESM1_ESM.pdf]

Optimization and Validation of Diffusion MRI-based Fiber Tracking with Neural Tracer Data as a Reference

Carlos Enrique Gutierrez[1*], Henrik Skibbe[2], Ken Nakae[3], Hiromichi Tsukada[1], Jean Lienard[1], Akiya Watakabe[4], Junichi Hata[5,9,10], Marco Reisert[6], Alexander Woodward[7], Yoko Yamaguchi[8,11,12], Tetsuo Yamamori[4], Hideyuki Okano[5,10], Shin Ishii[3], and Kenji Doya[1]

[1]Neural Computation Unit, Okinawa Institute of Science and Technology Graduate University, Okinawa, Japan
[2]Brain Image Analysis Unit, RIKEN Center for Brain Science, Wako, Japan
[3]Integrated Systems Biology Laboratory, Department of Systems Science, Graduate School of Informatics, Kyoto University, Kyoto, Japan
[4]Laboratory for Molecular Analysis of Higher Brain Function, RIKEN Center for Brain Science, Wako, Japan
[5]Laboratory for Marmoset Neural Architecture, RIKEN Center for Brain Science, Wako, Japan
[6]Department of Medical Physics, Medical Center, Freiburg University, Germany
[7]Connectome Analysis Unit, Riken Center for Brain Science, Wako, Japan
[8]Applied Electronics Laboratory, Kanazawa Institute of Technology, Japan
[9]Division of Regenerative Medicine, The Jikei University School of Medicine, Tokyo, Japan
[10]Department of Physiology, Keio University School of Medicine, Tokyo, Japan
[11]Graduate School of Information Science and Technology, The University of Tokyo, Tokyo, Japan
[12]Laboratory for Cognitive Brain Mapping, RIKEN Center for Brain Science, Wako, Japan
[*]carlos.gutierrez@oist.jp

Supplementary materials

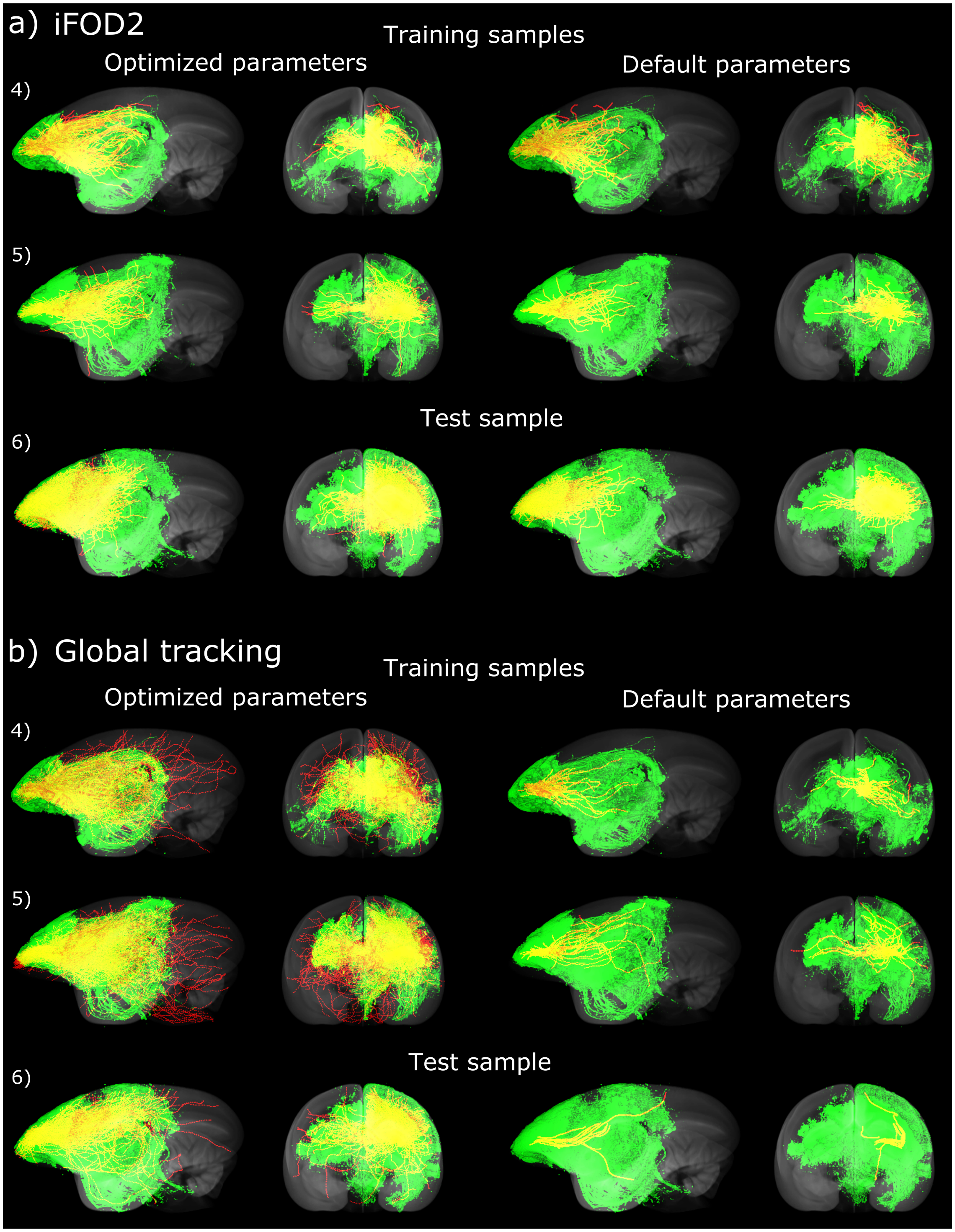

Figure S1. **Examples of tracked fibers by optimized and default parameters**. Unoccluded visualization of spatial relationships between fluorescent tracer signals (green) and tractography (red) for 3 injection sites: 4) and 5) from the training set; 6) from unseen marmoset subjects. Optimized results for both, a) iFOD2 and b) global tracking algorithms, show enlarged overlap (yellow) and longer fibers connecting sub-cortical and projection areas. Red fibers correspond to "false" positives. Figure created using FluoRender 2.24 (https://www.sci.utah.edu/software/fluorender.html) and Inkscape 1.0beta2 (https://inkscape.org/). Image datasets are part of the Brain/MINDS project (see Data availability section).

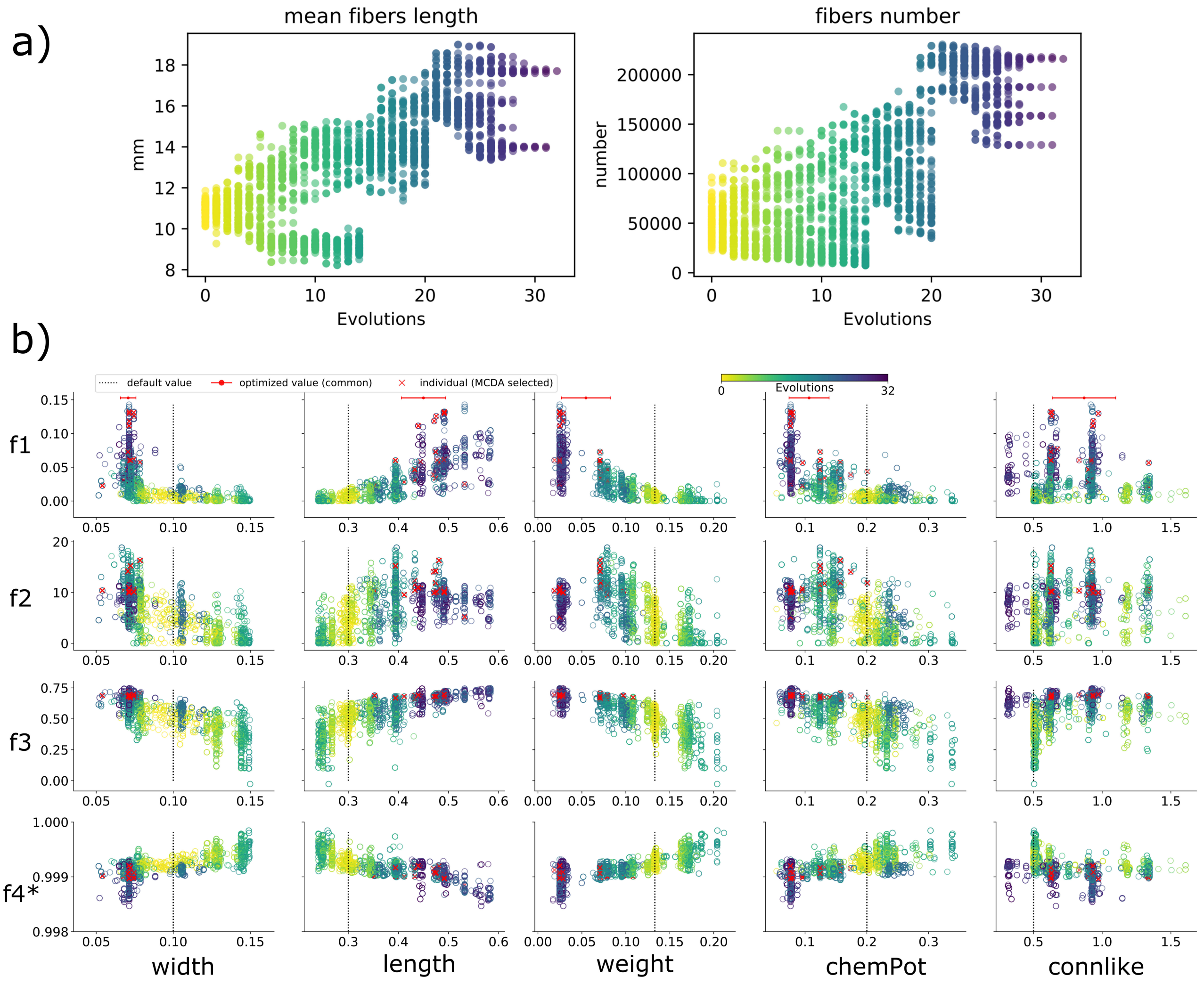

Figure S2. Parameter exploration and convergence to generic values for global tracking. a) Evolution of the number and mean length of fibers through optimization. b) Given the default parameter values (black dotted line) and exploration ranges, optimization (color coded) widely scrutinizes potential values to maximize objective functions. Different exploration paths are observed, mainly because of different brains. *width*, *weight* and *chemPot* show robustness to multiple brains by converging to similar values. The generic setting is computed as the mean and standard deviation (red dots at the top row) of the best scoring parameters (red x markers).

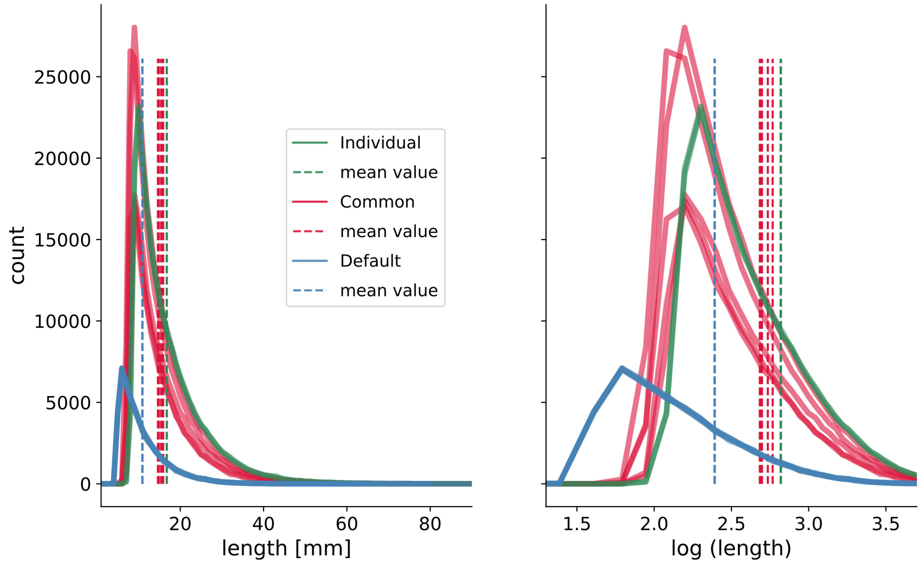

Figure S3. Fiber length performance. Fiber length histograms for a brain subject showing the default and optimized parameter results. 5 runs for the default and standard (common) parameters show the improvements, better displayed in the log-transformed histograms (right figure). Optimization increased considerable the number of fibers and extended their lengths. The mean fiber length (dashed lines) improved from around 10mm to 17mm. Common results are similar to the performance of the best setting (individual) found for the subject.

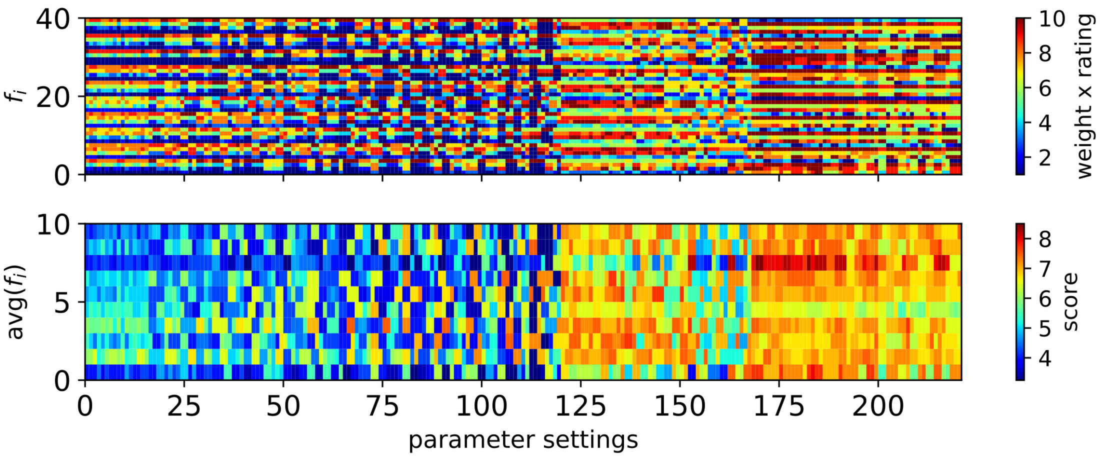

Figure S4. Multiple Criteria Decision Analysis (MCDA) on global tracking. Upper matrix rates parameter settings based on achieved objective values. The matrix below, averages the rates and calculates the final scores. Maximum scored settings per brain are selected as winners.

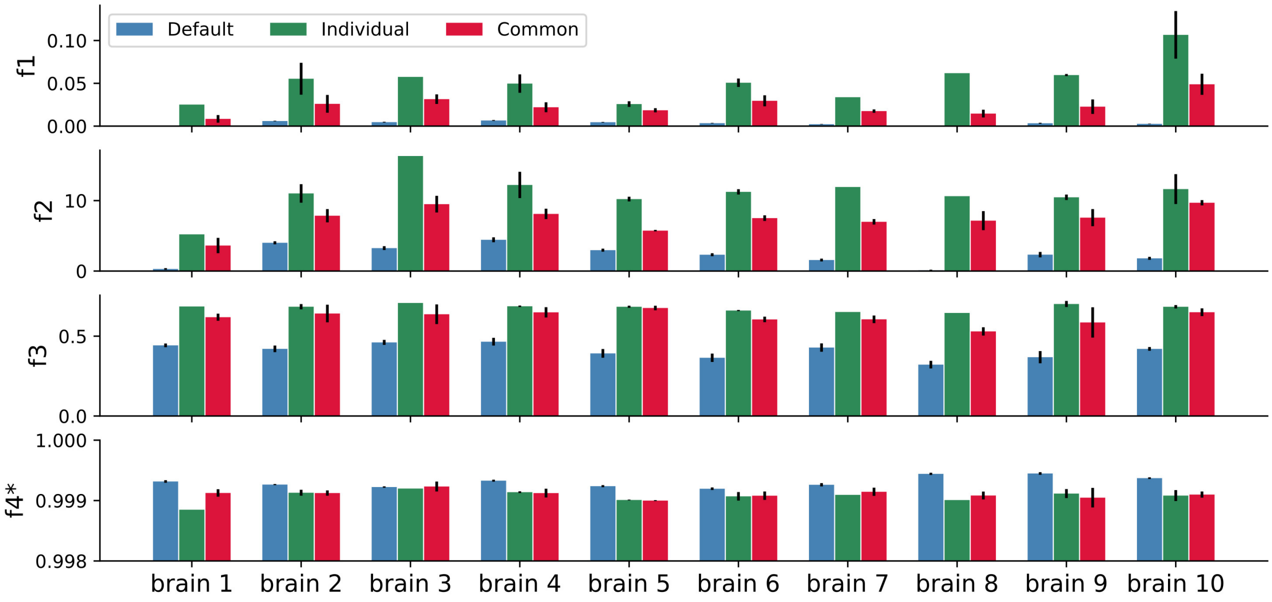

Figure S5. Training data set optimization results for global tracking. Objective function (average values for 5 runs) comparison of default, individual, and common (the latter two optimized) parameter settings for the training data set. Optimized parameters perform best at improving connections to projection areas f_3, while increasing coverage f_1 and true/false positive ratio f_2. Commissural passage f_4, expressed as the ratio of valid passage locations f_4^*, shows high accuracy.

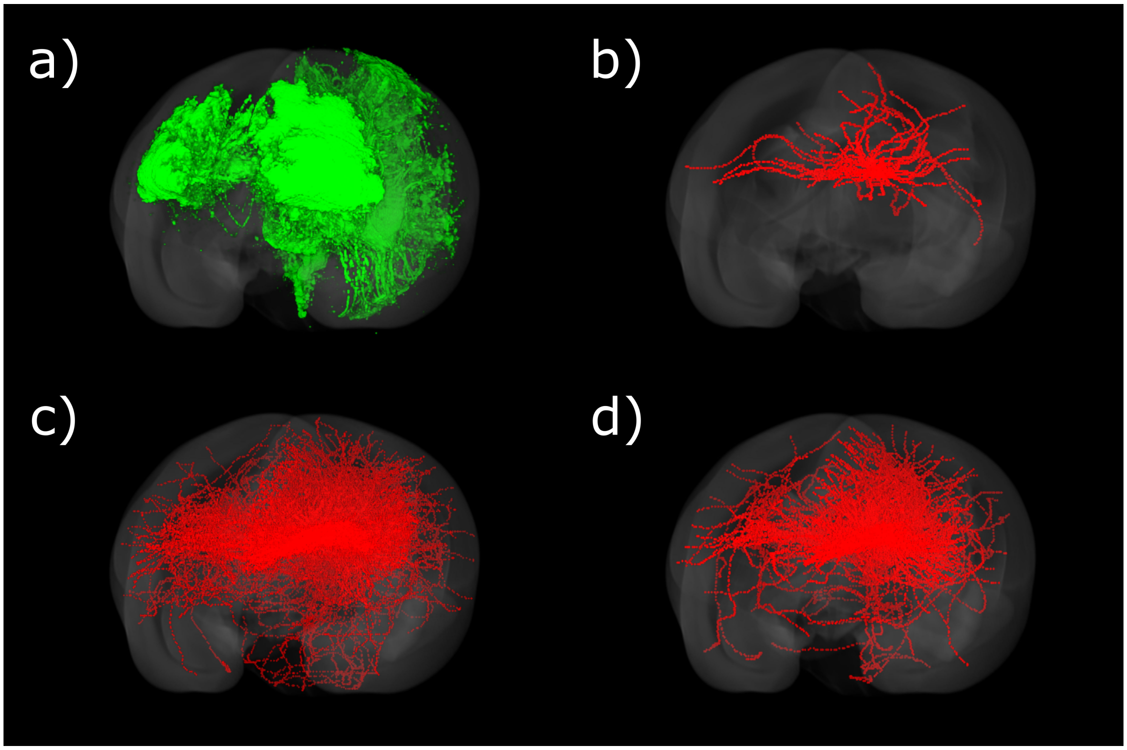

Figure S6. Optimized and default dMRI fiber tracking results. a) Neural tracer 3D reconstruction for a brain sample (green), and global tracking results (red). b) Fibers in contact with the injection region as a density map for the default parameters. c) Optimization improves fibers density map correspondence with the neural tracer for the individual setting. d) Standard parameters perform in a similar way to c), providing less dense results. Figure created using FluoRender 2.24 (https://www.sci.utah.edu/software/fluorender.html) and Inkscape 1.0beta2 (https://inkscape.org/). Image datasets are part of the Brain/MINDS project (see Data availability section).

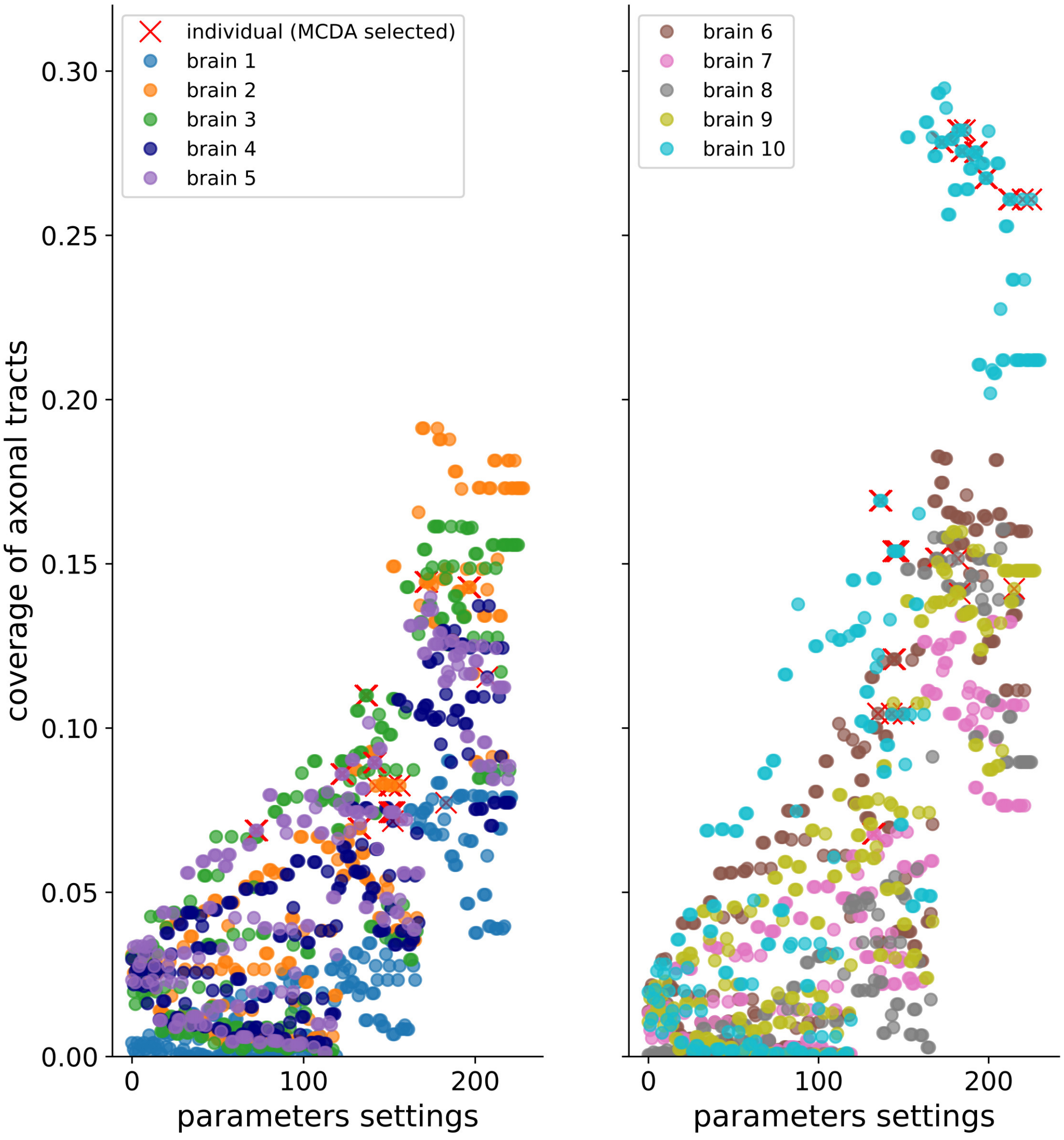

Figure S7. **Coverage of axonal tracts.** Performance of the strength-weighted coverage ($\sum_i^{N_{TP}} w_i / \sum_i^{N_P} w_i$) of axonal tracts (neural tracer) by global tracking fibers for the training set. Coverage improved in average from 0.9% (default settings) to 15% (winners settings, red x markers). A subject with less tracer volume (brain 10) obtained about 30% of coverage. The coverage values are affected by the high number of ”ground-truth” positive voxels and the mapping of fibers to high-resolution space.

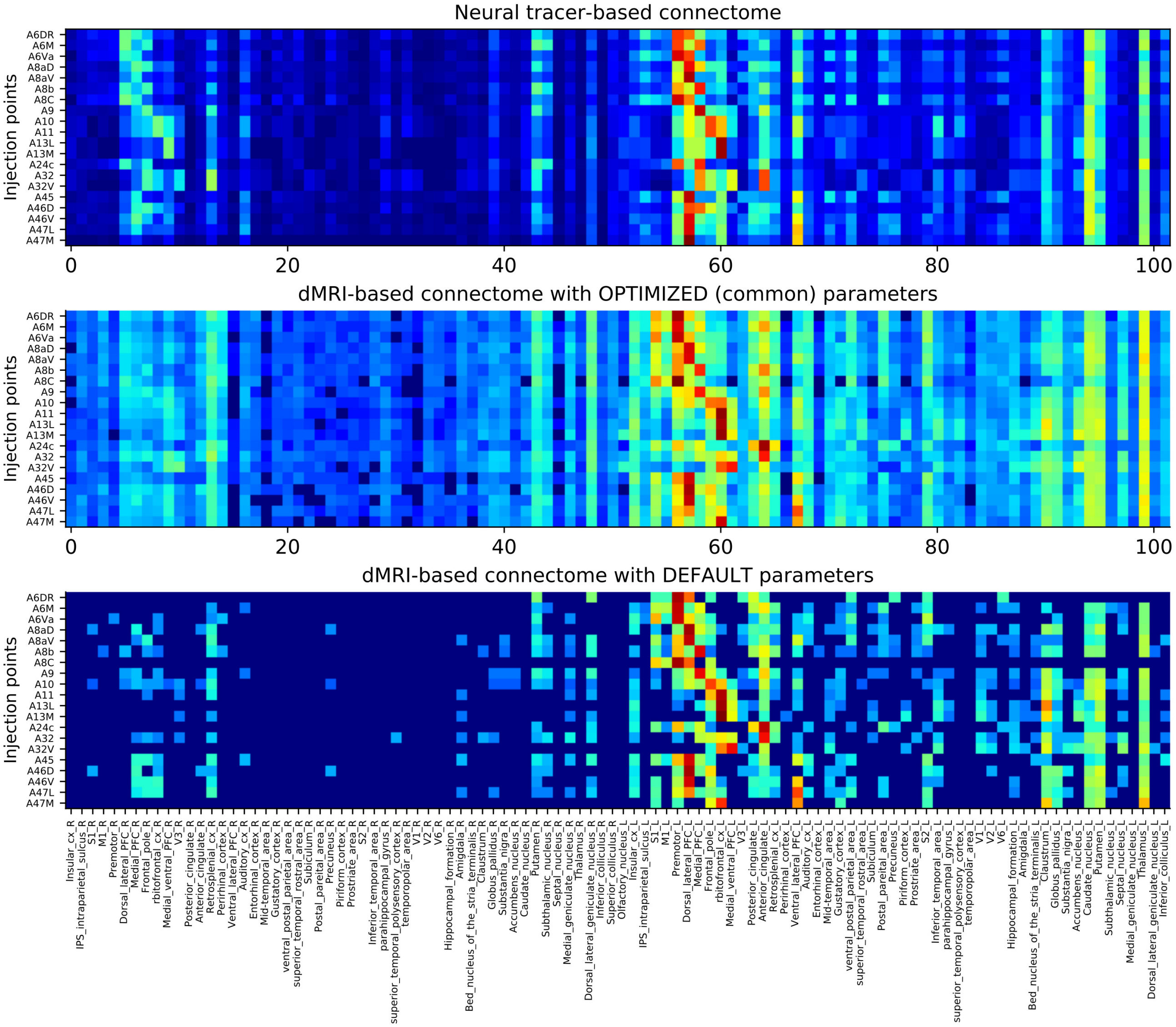

Figure S8. Brain-region level connectome comparison (global tracking). Neural tracer-based matrix (top) from marmonet displaying relative connection strengths from 20 injection regions in the marmoset pre-frontal cortex to the rest of the brain, organized in 104 parcellations. For the sake of comparison, optimized (center) and default (bottom) dMRI-based connectomes for a brain-subject are mapped to the same structure.

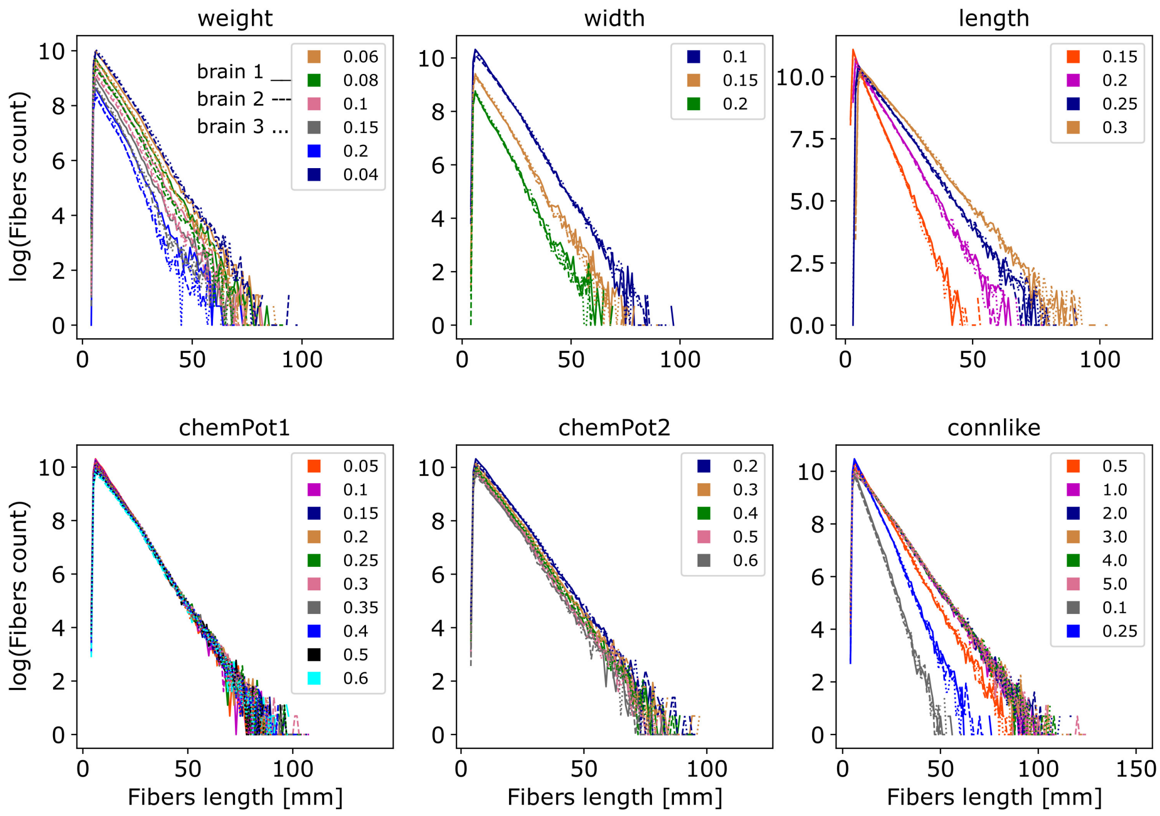

Figure S9. Global tracking parameters selection by relevance. Fiber length histograms for 3 marmoset brains (global tracking). Varying one parameter while maintaining fixed the others provides a clue about parameter's relevance. Bottom-left sub-plot shows almost no change for different values of *chemPot*1.
